# The Effects and Underlying Mechanisms of Hepatitis B Virus X Gene Mutants on the Development of Hepatocellular Carcinoma

**DOI:** 10.3389/fonc.2022.836517

**Published:** 2022-02-10

**Authors:** Rui Pu, Wenbin Liu, Xinyu Zhou, Xi Chen, Xiaomei Hou, Shiliang Cai, Liping Chen, Jianfeng Wu, Fan Yang, Xiaojie Tan, Jianhua Yin, Xin Wang, Guangwen Cao

**Affiliations:** ^1^ Department of Epidemiology, Second Military Medical University, Shanghai, China; ^2^ Department of Pathology, Xijing Hospital, Xi’an, China; ^3^ Laboratory of Molecular Cell Biology, Institute of Biochemistry and Cell Biology, Shanghai Institute for Biological Science, Chinese Academy of Science, Shanghai, China

**Keywords:** hepatitis B virus, mutation, hepatocarcinogenesis, PAI1, CDC20, inflammation

## Abstract

We aimed to elucidate the mechanism by which hepatitis B virus X (HBx) mutations increase the occurrence of hepatocellular carcinoma (HCC) and identify novel putative therapeutic targets. Wild-type HBx (WT-HBx) and four HBx mutants (M1, A1762T/G1764A; M2, T1674G+T1753C+A1762T/G1764A; M3, C1653T+T1674G+A1762T/G1764A; and Ct-HBx, carboxylic acid-terminal truncated HBx) were delivered into *Sleeping Beauty* (*SB*) mouse models. The HCC incidence was higher in the M3-HBx- and Ct-HBx-injected *SB* mice. M3-HBx had a stronger capacity of upregulating inflammatory cytokines than other HBx variants. Ectopic expression of M3-HBx and Ct-HBx significantly increased proliferation and S phase proportion of HepG2 and HeLa cells, compared to WT-HBx. Plasminogen activator inhibitor-1 (PAI1) and cell division cycle 20 (CDC20) were identified as novel effectors by cDNA microarray analysis. M3-HBx and Ct-HBx significantly upregulated the expression of PAI1 and CDC20 in HepG2 and HeLa cells as well as the livers of *SB* mice. Silencing PAI1 attenuated the effects of M3-HBx and Ct-HBx on the growth of HepG2 and HeLa cells. PAI1, an important player bridging the HBx mutants and HCC, should be a promising candidate as a prognostic biomarker and therapeutic target in HBV-related HCC.

## Introduction

Primary liver cancer (PLC) was the third leading cause of cancer death worldwide in 2020 and hepatocellular carcinoma (HCC) accounts for 75–85% of PLC cases (1). Chronic infection with hepatitis B virus (HBV) is the leading cause of HCC globally (2). Different countries have different rates of seropositivity for HBV surface antigen (HBsAg) in HCC (3). Approximately 85% of HCC cases in China are seropositive for HBsAg. HBV-related HCC (HBV-HCC) is associated with 10-year earlier onset, higher α-fetoprotein (AFP), and more microvascular invasion than HCC caused by other causes (4), indicating that HBV is more powerful in promoting HCC development than other etiological factors.

During HBV-induced hepatocarcinogenesis, HBV often evolves *via* accumulating its mutations adapted to the inflammatory microenvironment and integrating into the human genome (5, 6). HBV mutations, including A1762T/G1764A, C1653T, T1753V, and T1674G/C, in the core promoter (CP) region of the viral genome are typically the ones that increase the risk of HCC (7–9). It has been demonstrated that the combination (combo) mutant, rather than HBx with single or double CP mutations, accelerates cell cycle progression and p21 degradation, possibly *via* increasing expression of S-phase kinase-associated protein 2 (SKP2) (10). Gene expression profiling of human hepatocytes infected with HBV genotype F1b, particularly the CP mutant, indicates more cancer-related signaling pathways compared with other genotypes (8). HBV integration into the host genome often leads to the truncation of the HBV genome, particularly at the C terminus of hepatitis B virus X (HBx), resulting in the generation of carboxylic acid terminal-truncated HBV X protein (Ct-HBx) (11). The HBV X genes encoding Ct-HBx, which are most frequently detected in HBV-HCC samples, significantly increase the aggressiveness of HCC compared to the full-length X gene, possibly *via* downregulating thioredoxin-interacting protein (TXNIP), a well-established regulator of glucose metabolism (12). However, the gene expression profiles of the HBV mutant-integrated liver of animal models with intact immune system, which represents the key molecular event in humans, are not reported. Here, we investigated tumorigenic effects of combo HBx mutations (A1762T/G1764A, C1653T, T1753C, and T1674G), Ct-HBx, and wild-type (WT) HBx on HCC using the *Sleeping Beauty* (*SB*) transposon system to deliver WT-HBx and HBx mutants into the livers of fumarylacetoacetate hydrolase (*Fah*)-deficient mice. The functional genes with similar profiles in the animal model and cell lines were functionally investigated. This study not only helps elucidate the mechanisms by which HBx mutants promote HBV-induced carcinogenesis but also reveals effective therapeutic options for HBV-HCC.

## Materials and Methods

### Study Population

The effect of HBx combo mutation on the risk of HCC occurrence was evaluated with a cohort that was established in our previous study. In total, 2114 HBV-infected patients were enrolled from the second affiliated hospital of Second Military Medical University. The establishment of the cohort and detection of HBV mutations were as previously described (9). The last date of follow-up was 31 August 2019. The study protocol conformed to the 1975 Declaration of Helsinki and was approved by the ethics committee of Second Military Medical University. All participants provided written consent.

### Plasmid Construction

HBx fragments (nt. 1374–1838) were amplified from the sera of 10 HBV-infected patients included in the cohort. The details for viral DNA extraction, HBV DNA sequencing, and mutation analysis are provided in the **Supplementary Materials**. WT-HBx and the HBx mutants were linked with flag tags and inserted into *EcoRI* restriction sites of the pKT2-FAH-mCa-SB plasmid, an *SB* transposon vector containing the cDNA of *Fah* gene, respectively (13). The constructs were verified by Sanger sequencing. Primers for the amplification and verification are listed in **Table S1**. The construction of recombinant lentiviruses expressing WT-HBx and HBx mutants is described in the **Supplementary Materials**.

### Cell Experiments

HepG2 and HeLa cell lines were purchased from the Chinese Academy of Sciences (Shanghai, China). Before the experiments, all cell lines were authenticated using the genotyping analysis of short tandem repeat (STR) by Biowing Biotechnology (Shanghai, China). All cell cultures were tested for mycoplasma contamination every three months. The cells with the stable overexpression of HBx variants were constructed with lentivirus. Details are available in the **Supplementary Materials**. Small interfering RNAs (siRNAs) against cell division cycle 20 (*CDC20*), plasminogen activator inhibitor-1 (*PAI1*), and cyclin dependent kinase inhibitor 1A (*P21*) were synthesized by GenePharma (Shanghai, China) (**Table S2**). Lipofectamine 3000 kit (Invitrogen, Carlsbad, CA) was applied for siRNA transfection. Gene expression was measured by quantitative reverse transcription PCR (qRT-PCR) and Western blot (**Figure S1**). Cell proliferation, migration, invasion, cell cycle assay, qRT-PCR, and Western blot are detailed in the **Supplementary Materials**.

### Cell Proliferation, Migration, Invasion, and Cell Cycle Assay

Cell proliferation was assessed by the Cell Counting Kit-8 (CCK8) kit (Dojindo, Osaka, Japan). Cells were seeded into the 96-well plates (Corning Incorporated Coster, Kennebunk, ME). Then, 100uL of 10% CCK8-DMEM solution was added and the cells were incubated for 1.5 h. The number of cells was estimated by measuring optical density (OD450) for every 24 h. For colony formation assay, 500 cells were seeded into 6-well plates and were incubated for 21 days. Cell clones were stained with crystal violet and counted manually. The experiments were performed in triplicate.

The migration and invasion were measured using Transwell inserts (Corning, New York, NY). For the migration assay, a total of 1×10^4^ cells were placed in upper wells with 400µL serum-free DMEM. For the invasion assay, the upper chambers were coated with 20% Matrigel (BD, San Diego, CA) before cell seeding. The lower chamber was filled with 500 µL DMEM (10% FBS). After incubation for 48h, the migrated cells were digested and seeded into 96-wells plates. After incubation for 6h, 100µL DMEM containing 20% MTS (Promega, Madison, WI) and PMS (Promega) was added. After 2h incubation, absorbance at 490 nm was measured. Five fields were randomly selected and photographed with a microscope at 10 × magnification. Each assay was performed in triplicate.

The cell cycle assay was performed using flow cytometry (Merck Millipore, Rockville, MD). Cells were digested and fixed at -20°C in 70% ethanol overnight. The cell cycle distribution was measured by flow cytometry using the PI/RNase staining buffer (BD) and was analyzed by ModFit LT software (Verity Software House, Topsham, ME). Each assay was performed in triplicate.

### Mouse Models

The mouse model of HBx protein-induced HCC was constructed based on *Fah*-deficient mice (13). The *Fah^-/-^* mice were maintained with 7.5 μg/mL 2-(2-nitro-4-trifluoromethylbenzoyl)-1, 3-cyclohexanedione (NTBC). At the age of 5–7 weeks, mice were injected with 15μg constructs containing *Fah* cDNA and the HBx mutants or WT by hydrodynamic injection *via* tail vein. After the injection, NTBC was removed from the drinking water. Mice that died within 30 days after the injection were excluded from the following analyses due to the failure in gene delivery. All mice were sacrificed 150 days after the injection. The livers and tumors were pathologically examined. Hematoxylin–eosin (H&E) and immunohistochemistry (IHC) are detailed in the **Supplementary Materials**. The antibodies used for IHC are detailed in **Table S3**.

Five-week-old nude mice were purchased from Jihui Laboratory Animal Care Cooperation (Shanghai, China). Mice were subcutaneously injected with 1.5 × 10^6^ HeLa cells stably expressing wild-type or mutant HBx protein. Five weeks later, tumors were harvested. The use and care of animals were in compliance with institutional guidelines. All animal studies were conducted under the animal welfare protocol approved by the ethics committee of Second Military Medical University.

### HBV Capture Sequencing

Seven liver tissues and seven tumor tissues of the *SB* mouse models were subjected to HBV-capture sequencing. Liver tissues were from the mice without tumors, including three WT-HBx mice, one M3-HBx mouse, and three Ct-HBx mice. Tumor tissues were from one WT-HBx mouse, three M3-HBx mice, and three Ct-HBx mice. DNA was extracted from tissues using Genomic DNA Mini Kit (Invitrogen, Carlsbad, CA). The DNA libraries were constructed using the Fast Library Prep Kit (iGeneTech, Beijing, China). The capture probes of HBV panel were synthesized by iGeneTech. The libraries were captured using the probes of HBV panel and TargetSeq one enrichment kits (iGeneTech). The HiSeqTM 2500 platform (Illumina, San Diego, CA) was applied for the sequencing. The breakpoints of HBV integration were detected using VERSE software (14). The annotation for the integrated breakpoints was performed with ANNOVAR software (15). Data of HBV-capture sequencing were uploaded to the Sequence Read Archive (SRA) database under Accession Number PRJNA743432.

### Cytokine Assessment

The cytokine levels in mice plasma were measured by multiplex immunoassay with ProcartaPlex Kit (ThermoFisher Scientific, MA). In total, 11 cytokines were tested: interleukin-4 (IL-4), IL-5, IL-6, IL-1β, IL-12, interferon-α (IFNα), IFNγ, tumor necrosis factor-α (TNFα), granulocyte macrophage colony stimulating factor (GM-CSF), transforming growth factor β (TGFβ), and vascular endothelial growth factor (VEGF).

### Gene Expression Profiling and Functional Analysis

The gene expression profiles of HepG2 cells stably expressing WT-HBx and M3-HBx were analyzed by RNA-sequencing (RNA-seq). The sequencing libraries were constructed using TruSeq Stranded Total RNA with Ribo-Zero Gold Kit (Illumina, San Diego, CA). The HiSeqTM 2500 sequencing platform (Illumina, San Diego, CA) was applied for RNA-seq. HeLa cells were subjected to cDNA microarray assay by using Human Transcriptome Array 2.0 (Affymetrix, Santa Clara, CA). Three liver tissues from WT-HBx mice, three tumor tissues from M3-HBx mice, and three tumor tissues from Ct-HBx mice were also subjected to cDNA microarray analysis. The samples were randomly selected from each mouse group. Agilent-074809 SurePrint G3 Mouse GE V2.0 microarray (Agilent Technologies, Santa Clara, CA) was applied for this analysis. Mouse genes were converted to human homologs using the HUGO Gene Nomenclature Committee database. With the data of HepG2 cells, HeLa cells, and *SB* mice, differently expressed genes between WT-HBx group and M3-HBx group were identified using the edgeR package (16). The significantly expressed genes were subjected to gene set enrichment analysis (GSEA) online software (http://software.broadinstitute.org/gsea/). Based on the significantly enriched genes, PPI networks were generated using STRING v10 software (https://string-db.org/) (17). The gene profiling data HeLa cells and *SB* mouse models were uploaded to the Gene Expression Omnibus (GEO) database under Accession Numbers of GSE179126 and GSE179125, respectively. The sequencing data of HepG2 cells were uploaded to the SRA data base under Accession Numbers of SRP336293.

### Luciferase Reporter Assay

Promoter region was defined as 2000 bp upstream of the transcription start site of a given gene based on the Ensembl database (GRCh38.p13, https://asia.ensembl.org/Homo_sapiens). The promoter fragments of *CDC20*, *PAI1*, and *P21* were synthesized and sequenced by Obio Technology (Shanghai, China). The correct sequences were inserted into the firefly luciferase reporter vector (pGL4.10-basic vector, Promega) to evaluate the promoter activity. HepG2 and HeLa cells were co-transfected with the constructed reporter vectors and Renilla luciferase vectors (pRL-TK, Promega). Luciferase activities were tested using a dual-luciferase reporter assay system (Promega). Firefly luciferase activity was normalized to Renilla luciferase activity.

### Statistical Analysis

Differences in continuous variables were determined by Student’s *t*-test. For non-normal data, the Wilcoxon sum rank test was used. Hazard ratio (HR) and 95% confidence interval (CI) were estimated using the Cox regression analysis, adjusted for age and gender. The analyses of the data from *in vitro* and *in vivo* experiments were performed with GraphPad Prism version 5.0 (GraphPad Software, San Diego, CA). All statistical tests were two-sided*. P <* 0.05 was considered statistically significant.

## Results

### Effects of HBx Combo Mutations on the Risk of HCC Occurrence

**Figure 1:**
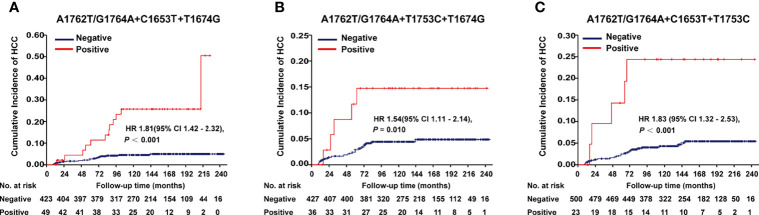
Effects of HBx combo mutations on HCC occurrence in a prospective cohort study with 2114 HBV-infected patients. **(A)** Effect of A1762T/G1764A+C1653T+T1674G on HCC occurrence. **(B)** Effect of A1762T/G1764A+T1674G+T1753C on HCC occurrence. **(C)** Effect of A1762T/G1764A+C1653T+T1753C on HCC occurrence. Hazard ratio (HR) was adjusted with age and gender.

A1762T/G1764A, T1674G, C1653T, T1674G, and T1753C were all identified as independent risk factors of HCC occurrence in our previous cohort study (9). In this cohort of HBV-infected patients, combo mutation A1762T/G1764A+C1653T+T1674G, A1762T/G1764A+T1674G+T1753C, and A1762T/G1764A+C1653T+T1753C were all significantly associated with an increased risk of HCC, with an age- and gender-adjusted HR (95% CI) of 1.81 (1.42-2.32), 1.54 (1.11-2.14), and 1.83 (1.32-2.53), respectively (**Figure 1**). The frequencies of combo mutations A1762T/G1764A+C1653T+T1674G, A1762T/G1764A+T1674G+T1753C, and A1762T/G1764A+C1653T+T1753C in the baseline sera of the HBV-infected population were 10.38%, 7.78%, and 4.4%, respectively. The combo mutation A1762T/G1764A+C1653T+T1674G and A1762T/G1764A+T1674G+T1753C were then selected for the following experiments because of higher frequencies in the HBV-infected population.

### Effects of WT-HBx and the HBx Mutants on Hepatocarcinogenesis in *SB* Mice

**Figure 2:**
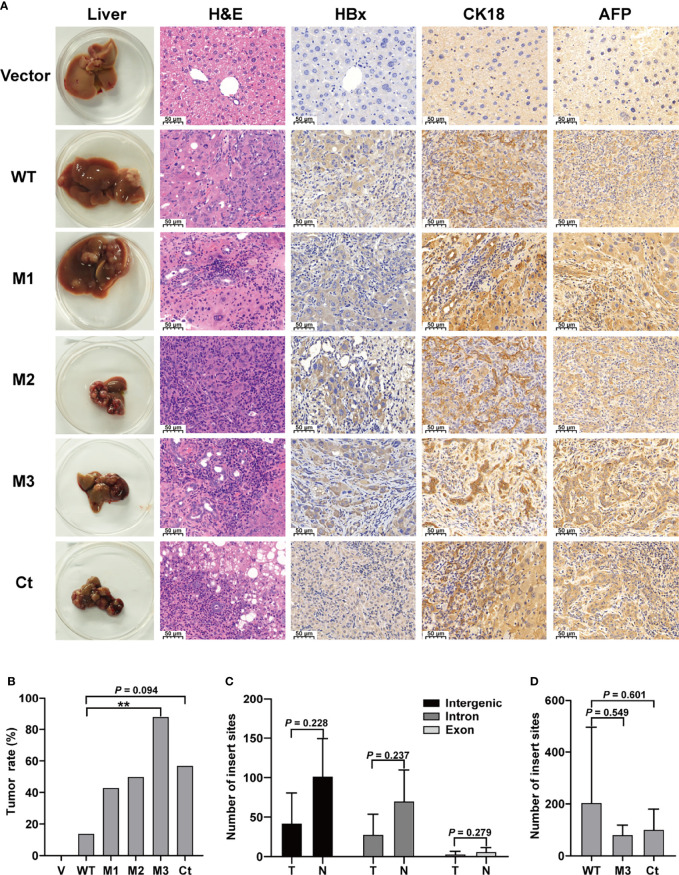
The pathological characteristics, tumor occurrence, and the insertion sites of HBx fragment in *Sleeping Beauty* (SB) mouse models. **(A)** Representative images of H&E and IHC staining. The first column shows representative gross features indicating the tumors in the livers. The second column shows representative H&E stainings indicating the histological features of the livers. The third to fifth columns show representative IHC stainings for HBx, CK-18, and AFP. Vector, the mice injected with the empty vector; WT, M1, M2, M3, and Ct, the mice injected with vectors carrying WT-HBx, M1-HBx, M2-HBx, M3-HBx, and Ct-HBx, respectively. **(B)** Incidence of tumors in the mouse models. V, the mouse models injected with the empty vector, n=7; WT, the mouse models injected with WT-HBx, n=7; M1, the mouse models injected with M1-HBx, n=7; M2, the mouse models injected with M2-HBx, n=4; M3, the mouse models injected with M3-HBx, n=8; Ct, the mouse models injected with Ct-HBx, n=7. **(C)** The number of HBx insertion sites in different functional regions and different tissue types. T, tumors from the mice with tumor nodules; N, liver tissues from the tumor-free mice. **(D)** The total number of HBx insertion sites between two of the three groups. **P < 0.01.

This study investigated four HBx mutants (nt.1374–1838) carrying the HCC-risk mutations: M1, A1762T/G1764A alone; M2, A1762T/G1764A+T1674G+T1753C; M3, A1762T/G1764A+C1653T+T1674G, and Ct-HBx (nt. 1374–1733) (**Figure S2**). *Fah^-/-^* mice were randomly assigned into 6 groups (vector, WT-HBx, M1-HBx, M2-HBx, M3-HBx, and Ct-HBx) with nine animals per group. Empty *SB* vector and those carrying WT-HBx and the HBx mutants were successfully delivered into the livers of 41 *Fah^-/-^* mice (vector, n = 8; WT-HBx, n = 7; M1-HBx, n = 7; M2-HBx, n = 4; M3-HBx, n = 8; Ct-HBx, n = 7). None of the empty vector-injected mice developed tumors. Tumors and shrunken and cirrhotic livers were evident in M2-HBx-, M3-HBx-, and Ct-HBx-injected mice (**Figure 2A**). No apparent pathological change was observed in the livers of the vector-injected mice. Inflammatory cell infiltration and cancer nests were observed in the HBx mutants-injected mice, which were similar to the histopathological changes of mouse hepatitis-induced HCC (18). Histopathologic analyses revealed that inflammatory cell infiltration was more severe in livers injected with HBx mutants than in those with WT-HBx (**Figure 2A**). Our IHC analysis confirmed that the tumors were positive for HBx protein as well as cytokeratin 18 (CK18) and AFP, the classic biomarkers of HCC (**Figure 2A**). The incidence of tumors was 14.3% (1/7), 42.8% (3/7), 50% (2/4), 87.5% (7/8), and 57.1% (4/7) in the *SB* models whose hepatic genomic DNA was integrated with WT-HBx, M1-HBx, M2-HBx, M3-HBx, and Ct-HBx, respectively. Compared to WT-HBx-injected mice, all HBx mutants-injected mice displayed a trend toward higher tumor burden, especially the M3-HBx and Ct-HBx groups (**Figure 2B**).

### The Insertion Sites of HBx Fragments

The *SB* system-induced integration sites were examined by HBV-capture sequencing in mice from the group of WT-HBx and two groups with highest HCC incidence, M3-HBx and Ct-HBx. In total, 1750 integrations were identified (**Table S4**). HBx fragments were mostly integrated into the intergenic and intron regions, with only 3.71% located in the exon regions. There were nine genes with the insertion sites detected in more than three samples (**Table S5**). The first three most frequently integrated genes were *Fah* (42.8%), catenin alpha 3 (*Ctnna3*) (28.6%), and fragile histidine triad gene (*Fhit*) (28.6%). Gene encoding telomerase reverse transcriptase (TERT) was not an integration site. The nine frequently integrated genes had low ratios of the reads with HBx integration to the reads of normal gene copies, ranging from 0.09% to 0.25%. The difference in the levels of integration sites was not significant between liver tissues and tumor tissues (**Figure 2C**). The number of integration sites was not significantly different among WT-HBx-, M3-HBx-, and Ct-HBx-injected *SB* mice (**Figure 2D**). These data suggest that the tumors developed in *SB* mice were induced by HBx proteins, rather than insertional mutations.

### Serum Levels of Proinflammatory Cytokine in WT-HBx and the HBx Mutants SB Mouse Models

**Figure 3:**
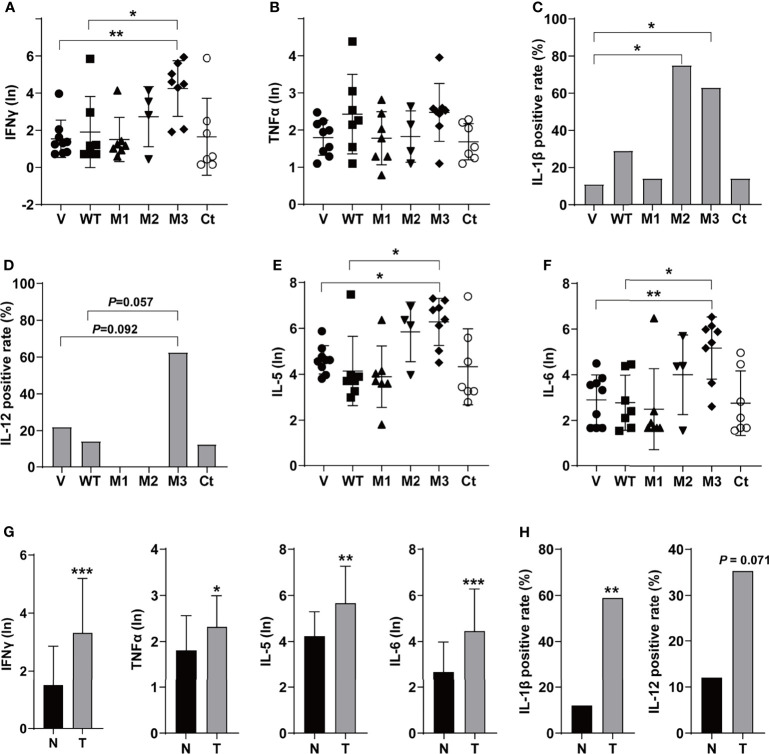
The serum levels and the positive rates of cytokines in the SB mouse models. **(A)** The serum level of IFN-γ; **(B)** the serum level of TNF-α; **(C)** the positive rate of IL-1β; **(D)** the positive rate of IL-12; **(E)** the serum level of IL-5; **(F)** the serum level of IL-6; **(G)** the serum levels of IFN-γ, TNF-α, IL-5, and IL-6 in the mouse models with or without tumor; and **(H)** the positive rates of IL-1β and IL-12 in the mouse models with or without tumor. N, the tumor-free mice; T, the mice with tumors. *P < 0.05; **P < 0.01; ***P < 0.001.

The levels of T helper 1 (Th1) cytokines (IFNγ, TNFα, IL-1β, and IL-12), Th2 cytokines (IL-4 and IL-5), IL-6, IFNα, TGFβ, VEGF, and GM-CSF were detected in the sera of the *SB* mice (**Figure 3** and **Figure S3**). Among the Th1 cytokines, IFNγ and TNFα were positive in all groups (**Figures 3A, B**). The level of IFNγ was significantly higher in the M3-HBx-injected mice than in the controls. The positivity rate of IL-1β was significantly higher in M2-HBx- and M3-HBx-injected mice than in the empty-vector-injected mice (**Figure 3C**). The M3-HBx-injected also showed a trend toward increased positivity rate of IL-12, compared with WT-HBx-injected mice (**Figure 3D**). IL-5 and IL-6 were measurable in all samples. The levels of IL-5 and IL-6 were significantly higher in M3-HBx mice than in the empty vector- and WT-HBx-injected mice (**Figures 3E, F**). The levels of IFNγ, TNFα, IL-5, and IL-6 were significantly higher in the mice with tumors than in the tumor-free mice (**Figure 3G**). The positive rate of IL-1β was significantly higher in the mice with tumors. Compared with tumor-free mice, the mice with tumors also showed a trend toward an increased positivity rate of IL-12 (**Figure 3H**). IL-4 was only positive in one Ct-HBx-injected mouse. IFNα was only positive in two M3-HBx-injected mice and one Ct-HBx-injected mouse (**Figure S3A**). The HBx mutants did not upregulate the expression of TGF-β, VEGF, and GM-CSF (**Figures S3B–D**). No significant difference was observed in the levels of VEGF, TGF-β, IFNα, and GM-CSF between the *SB* mice with tumors and the tumor-free *SB* mice (**Figures S3E, F**). Thus, compared with WT-HBx and other HBx variants, M3-HBx had a stronger capacity of upregulating IFNγ, IL-1β, IL-5, and IL-6, which play important roles in HCC development.

### Effects of the HBx Mutations on Malignant Phenotypes of Cancer Cells

**Figure 4:**
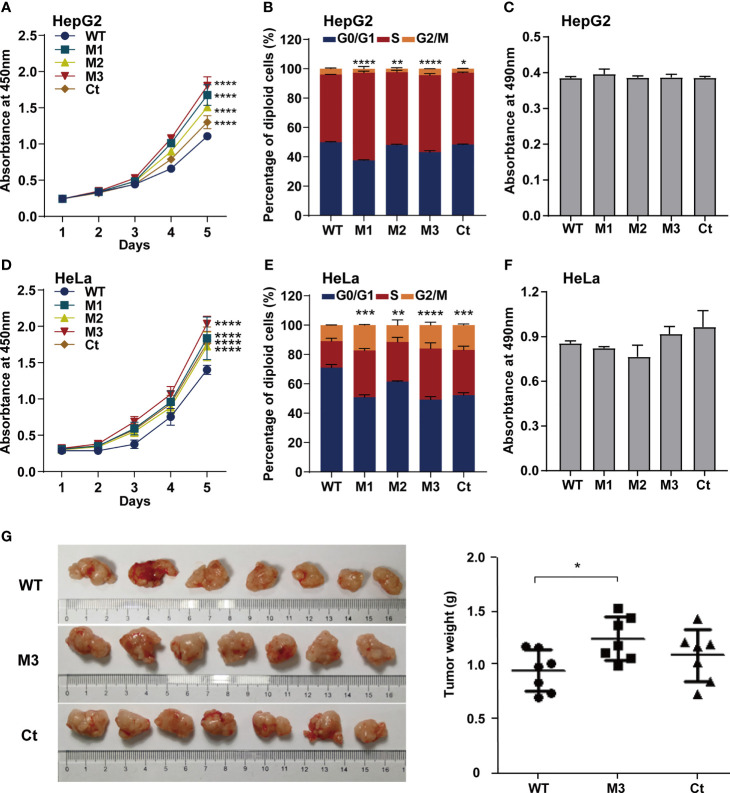
Effects of HBx mutations on malignant phenotypes of cancer cells. **(A)** CCK8 assays showed that ectopic expression of the HBx mutants significantly increased the proliferation of HepG2 cells, compared to WT-HBx. **(B)** Flow cytometry assays showed that the rate of cells in S phase was significantly higher in HepG2 cells expressing HBx mutants than in those expressing WT-HBx. **(C)** Transwell assays showed that ectopic expression of the HBx mutants had no significant effect on the migration of HepG2 cells, compared to WT-HBx. **(D)** CCK8 assays showed that ectopic expression of the HBx mutants significantly increased the proliferation of HeLa cells, compared to WT-HBx. **(E)** Flow cytometry assays showed that the rate of cells in S phase was significantly higher in HeLa cells expressing HBx mutants than in those expressing WT-HBx. **(F)** Transwell assays showed that ectopic expression of the HBx mutants had no significant effect on the migration of HeLa cells, compared to WT-HBx. **(G)** Tumor weights among different groups. *P < 0.05; **P < 0.01; ***P < 0.001; ****P < 0.0001.

To investigate whether the function of the HBx mutation was hepatocyte-specific, we evaluated the oncogenic effects of the HBx mutants in HepG2 and HeLa cells. The overexpression of WT-HBx and HBx mutants in HepG2 and HeLa cells *via* the lentiviral infection was confirmed by Western blot (**Figure S4A**). Ectopic expression of M1-HBx, M2-HBx, M3-HBx, and Ct-HBx significantly increased cell proliferation and the S phase proportion of HepG2, compared to WT-HBx (**Figures 4A, B**). No significant difference in migration and invasion was observed between HepG2 cells expressing WT-HBx and those expressing HBx mutants; the same was true for HeLa cells (**Figure 4C** and **Figures S4B–D**). The effects of HBx mutants on cell proliferation, cell cycle, and migration were repeated in HeLa cells (**Figures 4D–F**). As M3-HBx and Ct-HBx displayed the strongest tumorigenic capability in the *SB* mouse models, the effects of M3-HBx and Ct-HBx on the growth of cancer cells were further investigated using the xenograft of HeLa cells in nude mice (HepG2 is not tumorigenic in nude mice). It was found that the tumor weight was significantly higher in HeLa cells with M3-HBx than in those with WT-HBx (**Figure 4G**). Thus, the HBx mutants, especially M3-HBx and Ct-HBx, promote cancer development, and this effect is not hepatocyte-specific.

### HBx Mutants-Regulated Functional Molecules

**Figure 5:**
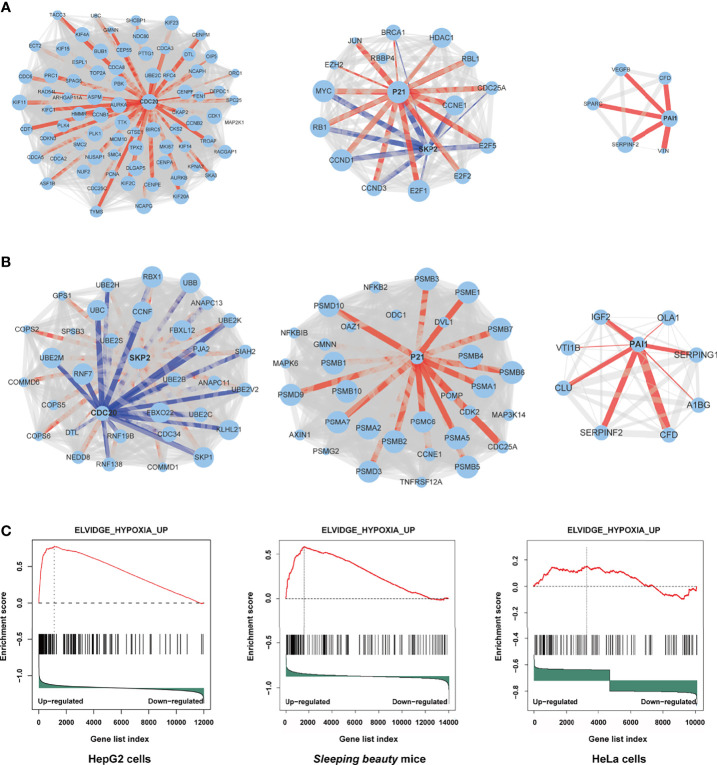
Independent protein-protein interaction (PPI) networks related to the introduction of M3-HBx and Ct-HBx into HepG2 cells by RNA-sequencing. **(A)** Three independent PPI networks from RNA sequencing data of M3-HBx-expressing HepG2 cells vs those of WT-HBx-expressing HepG2 cells. **(B)** Three independent PPI networks from RNA sequencing data of Ct-HBx-expressing HepG2 cells vs those of WT-HBx-expressing HepG2 cells. **(C)** The ELVIDGE_HYPOXIA_UP gene set including PAI1 was significantly enriched (false discovery rate < 0.05) in the M3-HBx-expressing HepG2 cells, the M3-HBx-expressing HeLa cells, and liver tissues of the M3-HBx-injected *SB* mice.

Through protein-protein interaction (PPI) analysis and gene sets enrichment analysis (GSEA), we identified the molecules playing important roles in the M3-HBx- and Ct-HBx-induced carcinogenesis. Based on RNA sequencing data of HepG2 cells, we identified the differentially expressed genes regulated by M3-HBx and Ct-HBx, compared to WT-HBx. PPI network analysis was performed with the differently expressed genes using the STRING algorithm (17). Three independent PPI networks were generated in M3-HBx group and Ct-HBx group, respectively. Four hub molecules with STRING enrichment scores >0.7 were identified, including PAI1, CDC20, P21, and SKP2 (**Figures 5A, B**). The findings were repeated in HeLa cells using cDNA microarray. PAI1, CDC20, P21, and SKP2 were also identified as functional molecules in the PPI networks of M3-HBx-expressing HeLa cells and Ct-HBx-expressing HeLa cells (**Figure S5A**). The cDNA microarray analysis was also performed in WT-HBx-, M3-HBx-, and Ct-HBx-injected mice. It was found that PAI1, P21, and SKP2 were significantly upregulated in both M3-HBx-injected mice and Ct-HBx-injected mice, compared to WT-HBx-injected control. CDC20 was only significantly upregulated in M3-HBx-injected mice (**Table S6**). The expression profiling data of the *SB* mouse models, HepG2 cells, and HeLa cells were subjected to GSEA analysis. Fifteen PAI1-involved gene sets were significantly enriched in the M3-HBx groups of both HepG2 cells and the *SB* mice. Among them, ELVIDGE_HYPOXIA_UP (19) was also significantly enriched in the M3-HBx group of HeLa cells (**Figure 5C**). Seven CDC20-related, 3 SKP2-related, and 2 P21-related gene sets were significantly enriched in the M3-HBx groups of both HepG2 cells and the *SB* mice (**Table S7**). None of these gene sets was repeated in HeLa cells. A chemotherapy-related gene set, KERLEY_RESPONSE_TO_CISPLATIN_UP (20), including P21 and PAI1 was significantly enriched in the Ct-HBx groups of both HepG2 cells and the *SB* mice. Compared to other hub molecules, PAI1 was involved in more gene sets related to the common mechanism of M3-HBx- and Ct-HBx-induced carcinogenesis. The IL-6-related gene set, CROONQUIST_IL6_DEPRIVATION_DN (21), was significantly enriched in the M3-HBx- and Ct-HBx-injected *SB* mice (**Figures S5B–C**). In total, 71 genes were included in this IL-6-related gene set. Interestingly, of the 71 genes, 58 (81.69%) were also included in the cancer-related gene sets enriched in M3-HBx groups of both HepG2 cells and the *SB* mice. A previous study has demonstrated that the expression of TXNIP is down-regulated by the Ct-HBx transfection (12). Therefore, the effect of HBx mutation on the expression of TXNIP was then investigated with the expression profiling data of HepG2 cells and the *SB* mice. Compared to WT-HBx, Ct-HBx significantly downregulated the mRNA level of TXNIP in HepG2 cells (change fold = 0.54, *P* = 0.003). However, this effect was unable to be repeated in the *SB* mice. M3-HBx showed no obvious effect on the expression of TXNIP either in the cell cultures or in the *SB* mice.

### Effects of the HBx Mutants on the Expression of Functional Molecules

**Figure 6:**
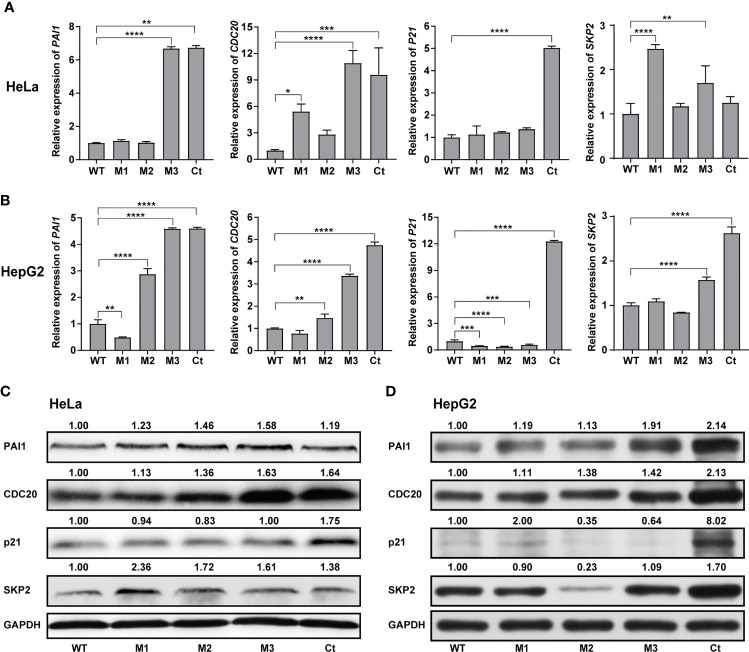
Effects of the HBx mutants on the expression of functional molecules *in vitro.* **(A)** Ectopic expression of Wt-HBx or HBx mutants affects the mRNA levels of *PAI1*, *CDC20*, *P21*, and *SKP2* in HeLa cells. **(B)** Ectopic expression of Wt-HBx or HBx mutants affects the mRNA levels of *PAI1*, *CDC20*, *P21*, and *SKP2* in HepG2 cells. **(C)** Ectopic expression of Wt-HBx or HBx mutants affects the protein levels of PAI1, CDC20, P21, and SKP2 in HeLa cells. **(D)** Ectopic expression of Wt-HBx or HBx mutants affects the protein levels of PAI1, CDC20, P21, and SKP2 in HepG2 cells. *P < 0.05; **P < 0.01; ***P < 0.001; ****P < 0.0001.

**Figure 7:**
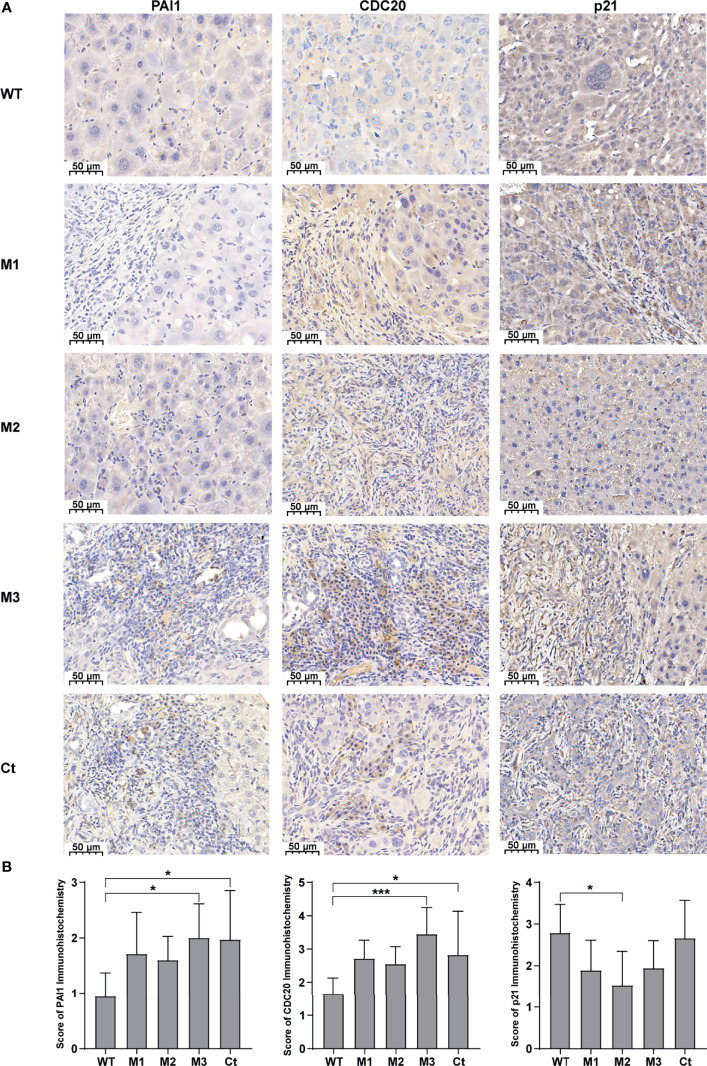
The expression levels of PAI1, CDC20, and p21 in the mouse tumor tissues of the SB mouse models injected with WT-HBx or each of the four HBx mutants. **(A)** Representative IHC images of PAI1 staining, CDC20 staining, and p21 staining. **(B)** Histograms of the IHC scores for the protein expression of PAI1, CDC20, and p21. *P < 0.05; ***P < 0.001.

We then investigated the expression levels of the above hub molecules in HepG2 and HeLa cells stably expressing WT-HBx and HBx mutants, respectively. The mRNA and protein levels of PAI1 and CDC20 were significantly upregulated by M3-HBx and Ct-HBx in HepG2 and HeLa cells (**Figures 6A–D**). All HBx combo mutations (M1-HBx, M2-HBx, and M3-HBx) significantly downregulated the mRNA level and protein level of p21 in HepG2 rather than in HeLa cells (**Figures 6B, D**). However, Ct-HBx significantly upregulated the expression of p21 in both HepG2 and HeLa cells. At the protein level, Ct-HBx only upregulated the expression of SKP2 in HepG2 cells. The HBx mutants showed consistent effects on the RNA and protein expression of PAI1, CDC20, and p21, respectively. However, our luciferase assays showed that M3-HBx and Ct-HBx had no direct effects on the promoter activities of the three molecules, compared with WT-HBx in HepG2 and HeLa cell lines (**Figure S6**). We then investigated the expression of PAI1, CDC20, and p21, the newly identified molecules participating in HBx-induced carcinogenesis, in the tumor tissue of the *SB* mice. The protein levels of CDC20 and PAI1 were significantly higher in the tumors from M3-HBx and Ct-HBx-injected mice, compared to their expression in the tumors from WT-HBx-injected mice (**Figures 7A, B**). The protein level of p21 in the tumors was significantly lower in the M2-HBx group than in the WT-HBx group.

### Reversal of HBx Mutations’ Effects by Silencing Functional Molecules

**Figure 8:**
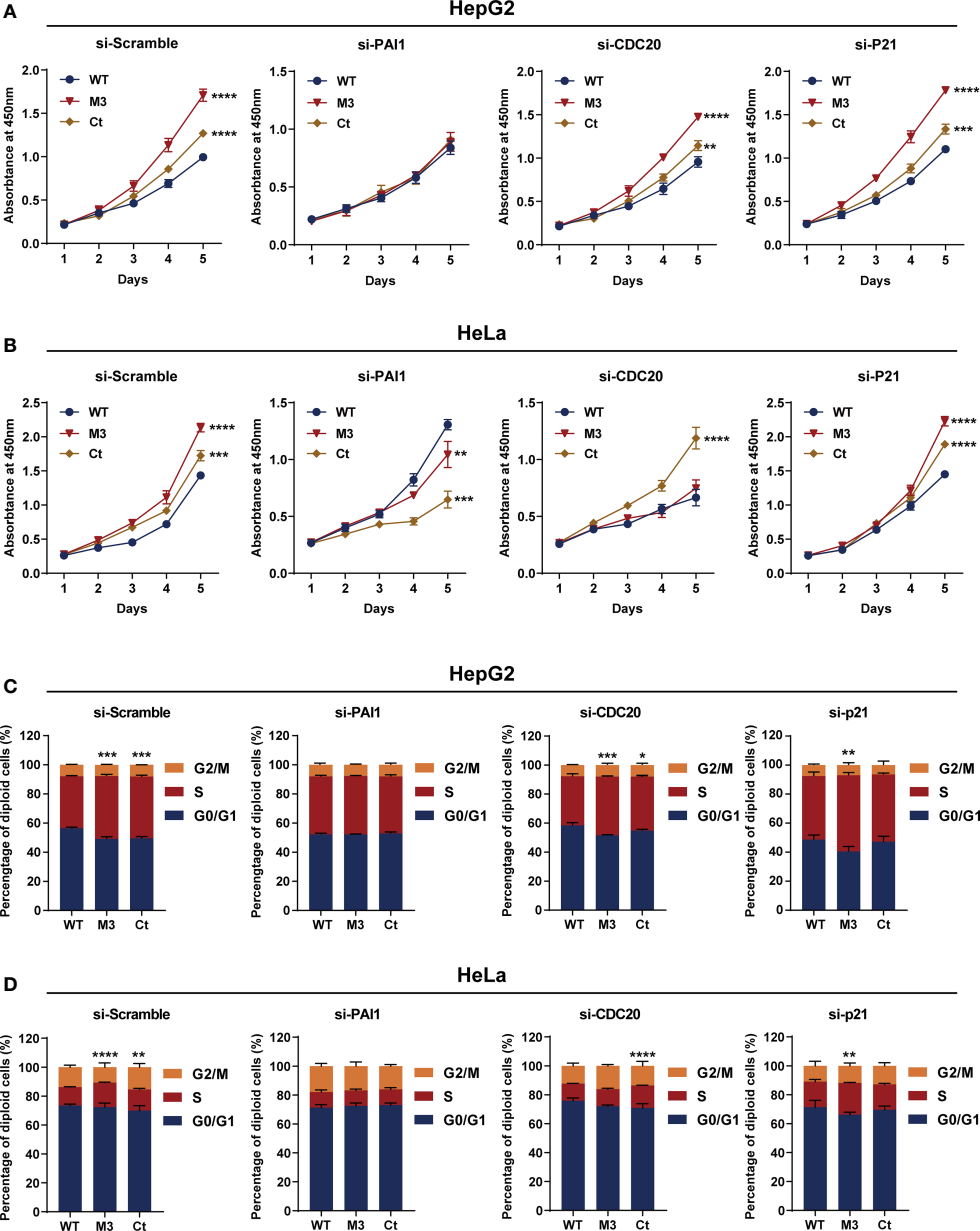
Silencing functional molecules influences the oncogenic effects of the HBx mutants. **(A)** Silencing PAI1, CDC20, and p21 influences the effects of HBx mutants on the proliferation of HepG2 cells. **(B)** Silencing PAI1, CDC20, and p21 influences the effects of HBx mutants on the proliferation of HeLa cells. **(C)** Silencing PAI1, CDC20, and p21 influences the effects of HBx mutants on the cell cycle in HepG2 cells. **(D)** Silencing PAI1, CDC20, and p21 influences the effects of HBx mutants on the cell cycle in HeLa cells. WT-HBx served as a control group in all comparisons. *P < 0.05; **P < 0.01; ***P < 0.001; ****P < 0.0001.

Among the four bioinformatics analysis-demonstrated hub molecules, SKP2 was previously identified to be involved in HBx-induced carcinogenesis (10). To figure out if the HBx mutations regulate the biological activity of HepG2 and HeLa cells *via* upregulating PAI1, CDC20, and p21, we knocked down the three genes and then examined the effect of stably expressed WT-HBx and HBx mutants on the proliferation of HepG2 and HeLa cells. PAI1 silencing significantly attenuated the effects of M3-HBx and Ct-HBx on the proliferation and cell cycle of HepG2 and HeLa cells. CDC20 silencing significantly reversed the effects of M3-HBx on the proliferation and cell cycle in HeLa cells. P21 silencing reversed the Ct-HBx-induced increase in the proportion of S phase cells, rather than cell proliferation in HeLa and HepG2 cells (**Figure 8**).

## Discussion

In this study, we found that the HBx mutants-injected mice, especially the M3-HBx- and Ct-HBx-injected mice, displayed a trend toward higher tumor burden, than the WT-HBx counterpart. This result is consistent with a previous study demonstrating that the HBx with A1762T/G1764A (K130M/V131I) mutation (the same as M1-HBx in our study) had a stronger tumorigenic effect than its WT counterpart in a *SB* mouse model (22). M2-HBx and M3-HBx contain two more HCC-risk mutations (7–9). The CP region of HBV genome harbors a complex spectrum of viral mutations. Compared to Ct-HBx, the oncogenic effect and underlying mechanisms of HBx combo mutations were rarely investigated. We provide evidence supporting the oncogenic function of T1674G+T1753C+A1762T/G1764A (M2) and C1653T+T1674G+A1762T/G1764A (M3) combo mutations. M3-HBx- and Ct-HBx-injected mice developed more tumors than did M1-HBx-injected *SB* mice. As WT-HBx and the HBx mutants were integrated into the hepatic genome, HCC development can be caused by the intergrated genes such as TERT (12, 23, 24). We then applied HBV-capture sequencing to identify the integration sites. TERT was not identified as the target. In this study, HBx was inserted into mouse genome through the *SB* system that is different from the mechanism by which HBV integrated into host genome during chronic infection. Therefore, the insertion sites of HBx in the *SB* mice were different with the insertion sites detected in human genome (23). The number of HBx insertion sites was similar in tumor and normal livers of the *SB* mice, suggesting that the difference in gene expression between tumor and normal livers was not induced by HBx insertion. The introduced WT-HBx or mutant HBx genes express HBx protein, as detected by IHC (**Figure 2A**). The tumors were confirmed to be HCC as CK-18 and AFP were positive (25). Thus, HCC developed in the *SB* mice are induced by the HBx proteins, rather than the insertional mutations. To further determine the effects of HBx mutants on the growth, we introduced WT-HBx and the HBx mutants into HepG2 and HeLa cells, respectively. Ectopic expression of HBx mutants significantly increased cell proliferation and the S phase proportion. Thus, the HBx mutants are more carcinogenic than WT-HBx. Of those, M3-HBx and Ct-HBx are the most potent ones.

Our histopathologic analyses revealed more inflammation events in livers of the mice injected with M2-HBx, M3-HBx, and Ct-HBx than their corresponding WT-HBx counterparts and even M1-HBx; furthermore, signs of necrosis, fibrosis, and degenerative changes were also evident in liver sections (**Figure 2A**). The serum levels of IFNγ, IL-5, and IL-6 were significantly higher in M3-HBx-injected mice than in the empty vector- or WT-HBx-infected controls, while the positive rate of IL-1β was significantly higher in M2-HBx- and M3-HBx-injected mice than in the vector-injected mice (**Figure 3**). IFNγ has both protumor and antitumor activities, although it induces the Th1-recruiting, proinflammatory chemokines in the tumor microenvironment (26). IL-5 contributes to the differentiation and survival of eosinophils in the tumor microenvironment, which facilitates tumor progression (26, 27). IL-6 promotes cancer immune evasion and facilitates the development of HCC (28). IL-1β, a pro-inflammatory cytokine abundantly expressed in the tumor microenvironment, plays a major role in cancer invasiveness, progression, and metastases. Anti-inflammation with canakinumab targeting IL-1β significantly reduces cancer occurrence and mortality (29). The higher circulating levels of IFN-γ, IL-5, and IL-6 and higher positivity rate of IL-1β are quite consistent with the higher occurrence of HCC in M3-HBx-injected mice, indicating M3-HBx-caused inflammation is closely related to HCC development. The IL-6-related gene set was significantly enriched in M3-HBx- and Ct-HBx-injected *SB* mouse models. In this gene set, 58 (81.69%) genes were also included in the cancer-related gene sets enriched in both HepG2 cells and the *SB* mice. These data indicate that the inflammatory factors, especially IL-6, play key roles in the development of HBx mutants-caused HCC.

Ectopic expression of the HBx mutants significantly increased cell proliferation and the S phase proportion of HepG2. These effects were exactly repeated in HeLa cells. Thus, the effects of the HBx mutants on cell biology are not hepatic-specific. HBV hepatotropism is mediated by specific receptor recognition in the liver (30). After being introduced, the HBx mutants exhibit pro-carcinogenic effects in non-hepatic cells such as HeLa cells (31). In this study, we found that Ct-HBx inhibited the transcription of TXNIP, a negative regulator of glucose uptake, in HepG2 cells. Furthermore, M3-HBx showed no obvious effect on the expression of TXNIP in HepG2 cells. Interestingly, full-length HBx can increase the level of free TXNIP without directly regulating the transcription of TXNIP (32); whereas, Ct-HBx has been proven to down-regulate the expression of TXNIP significantly in hepatic cell lines, compared to WT-HBx (12). These evidences imply that the 3’-terminal of HBx might facilitate the expression of *TXNIP via* binding to its putative promoter. As TXNIP suppression enhances glycolysis and facilitates the adaptation to the hypoxic environment (12, 33), Ct-HBx might increase glycolysis and facilitates the adaptation of HCC to the hypoxic environment. In the PPI networks, we identified four key molecules (PAI1, CDC20, P21, and SKP2) whose expressions were dysregulated the HBx mutants in the two human cancer cell lines. ELVIDGE_HYPOXIA_UP, the gene set including PAI1 was significantly enriched in the expression profiles of the tissues of SB mice and the two human cell lines. Thus, the PAI1-related alteration of energetic metabolism may function as a common mechanism by which HBx mutation promotes cancer evolution. The expression levels of SKP2 and p21 in HepG2 cells with M3-HBx are quite consistent with a previous study (10), indicating that our experiments are reliable. As a well-known tumor suppressor, p21 acts paradoxically *via* promoting the activation of cell cycle and tumor growth. Although p21 usually functions as a downstream molecule of p53 and inhibits cyclin-dependent kinase (CDK), p21 also facilitates the formation of cyclin-CDK complexes in a p53-independent manner (34). The effect of p21 on oncogenesis is also affected by subcellular localization. Nuclear p21 inhibits whereas cytoplasmic p21 promotes cell proliferation (35). HBx has been proven to promote cell cycle progression and cell proliferation by upregulating cytoplasmic p21 (36). No study reports the effect of Ct-HBx on p21 expression. Here, we found that the expression level of p21 was up-regulated by transfected Ct-HBx in HepG2 and HeLa cells, compared to the WT-HBx, possibly because the 3’-terminal of HBx inhibits the expression of p21 in cancer cells. We found that p21 knockdown reversed the positive effect of Ct-HBx on the proportion of S phase cells, indicating that p21 mediates the effect of Ct-HBx on cell cycle. The effects of the HBx mutants on CDC20 expression are also not reported. CDC20 is an activator of the anaphase-promoting complex/cyclosome. Recent studies have demonstrated that HBx induces mitotic checkpoint dysfunction *via* targeting the human serine/threonine kinase BubR1, while HBx binding to BubR1 attenuates the association between BubR1 and CDC20 (31, 37). Ct-HBx and HBx mutations at the Kunitz domains may fail to bind BubR1, thus facilitating the binding of hBubR1 to CDC20. However, this could not explain why Ct-HBx and M3-HBx upregulated the expression of CDC20 in HepG2 and HeLa cells and in the liver of corresponding SB mice. The reason that the effect of M3-HBx on the growth of HeLa cells is attenuated by silencing CDC20 remains to be elucidated. The mechanisms by which the HBx mutants upregulated the expression of the four key molecules remain unknown. HBx was proved to upregulate gene transcription by directly binding promoter, promoting the formation of transcription factor complex, or mediating regional demethylation of CpG islands (38). Our reporter assays indicated that the HBx mutants do not *trans*-activate the promoter activities of *PAI1*, *CDC20*, and *P21*. It is possible that the HBx mutants *trans*-activate their enhancers or change the chromatin accessibility *via* epigenetic modifications.

PAI1 (also known as SERPINE1) is a multifaceted proteolytic factor that not only has anti-fibrinolytic and anti-plasminogen activation activities but also plays a pro-tumorigenic role *via* its pro-angiogenic and anti-apoptotic activities (39). In the tumor microenvironment, PAI1 expression is upregulated in tumor-associated macrophages after being stimulated with cancer-associated fibroblasts-generating CXCL12 and promotes the malignant behavior of the HCC cells by mediating epithelial–mesenchymal transition (40). High levels of PAI1 and its genetic predisposition have been consistently associated with unfavorable prognosis in several types of human cancers including HBV-HCC (40–43). However, the interaction between PAI1 and HBx was rarely investigated. A previous study reported that WT-HBx has no effect on the expression of PAI1 (44). For the first time, we found that the expression of PAI1 was significantly upregulated by Ct-HBx and M3-HBx in cell lines and in the *SB* mice. Both M3-HBx and Ct-HBx alter the PAI1 related hypoxia pathway. Silencing PAI1 attenuates the positive effect of M3-HBx or Ct-HBx on the growth and the G1 to S transition of HepG2 cells. PAI1 plays important roles in lipid metabolism and glucose metabolism (45). Increased level of PAI1 was proved to promote cell cycle progression in pancreatic cancer (46). Thus, the HBx mutants upregulate the expression of PAI1, facilitating the generation of the cancer-promoting microenvironment in affected livers, promoting the metabolism reprogram, enhancing the cell cycle progression, and consequently contributing to the hepatocarcinogenesis. Thus, PAI1 is a promising candidate as a predictive and/or prognostic biomarker and a therapeutic target in HBV-HCC, especially for patients carrying HBx mutations.

Our study has limitations. First, we did not elucidate the mechanism by which the HBx mutants upregulate the expression of PAI1, CDC20, and p21, although we exclude the possibility that the HBx mutants *trans*-activate their promoters. Second, other mechanisms such as Ct-HBx promotes the development of HCC caveolin-1/low density lipoprotein receptor related protein 6/β-catenin/FERM domain containing 5 axis and deregulates centrosome-microtubule dynamics (47, 48) were not investigated. Third, the mechanisms by which M2-HBx, M3-HBx, and Ct-HBx induced cancer-promoting inflammation remain to be investigated.

## Conclusion

The HBx combo mutation C1653T+T1674G+A1762T/G1764A promotes carcinogenesis *via* upregulating the expression of PAI1 and CDC20 and inducing cancer-promoting inflammation. The oncogenic effect of Ct-HBx also depends on the upregulation of PAI1. Thus, PAI1 should be an important predictive and prognostic biomarker and promising therapeutic target in HBV-HCC.

## Data Availability Statement

The datasets presented in this study can be found in online repositories. The names of the repositories and accession numbers can be found in the article.

## Ethics Statement

The animal study was reviewed and approved by the ethics committee of Second Military Medical University.

## Author Contributions

GC: Conceptualization, Supervision, Writing - Review & Editing, Project administration. RP, XZ, XH, SC, LC, JW, and FY: Investigation, Validation. XC: Software, Formal analysis. WL and XZ: Formal analysis, Visualization, Writing - Original Draft. XW: Resources. XT and JY: Methodology. All authors read and approved the final manuscript.

## Funding

This work was supported by grant 2015CB554006 from the National Key Basic Research Program of China (GC); grants 91529305 (GC), 81520108021 (GC), 81673250 (GC), 81521091 (GC), 82003538 (WL), and 81502882 (XC) from the National Natural Science Foundation of China, grant 20YF1458800 (WL) from the Shanghai Yangfan Program; and grants GWV-10.1-XK17 from the “3-year public health promotion” program of Shanghai Municipal Health Commission (GC).

## Conflict of Interest

The authors declare that the research was conducted in the absence of any commercial or financial relationships that could be construed as a potential conflict of interest.

## Publisher’s Note

All claims expressed in this article are solely those of the authors and do not necessarily represent those of their affiliated organizations, or those of the publisher, the editors and the reviewers. Any product that may be evaluated in this article, or claim that may be made by its manufacturer, is not guaranteed or endorsed by the publisher.

## Supplementary Material

Supplementary Figure 1 The efficiency of gene knockdown. **(A)** The mRNA and protein levels of CDC20, PAI1, and p21 in HepG2 cells. **(B)** The mRNA and protein levels of CDC20, PAI1, and p21 in HeLa cells. ****P <* 0.001.

Supplementary Figure 2 The sequences of HBx fragments amplified from the peripheral serum samples of HBV-infected patients. WT, the HBx fragment without HCC-related mutations; M1, the HBx fragment carrying A1762T/G1764A; M2, the HBx fragment carrying combo mutation A1762T/G1764A+T1674G+T1753C; M3, the HBx fragment carrying combo mutation C1653T+T1674G+A1762T/G1764A; Ct, the fragment of carboxylic acid-terminal truncated HBx.

Supplementary Figure 3 The serum levels and the positive rates of cytokines in the SB mouse models. **(A)** The serum level of VEGF. **(B)** The serum level of TGFβ. **(C)** The positive rate of IFNα. **(D)** The positive rate of GM-CSF. **(E)** The serum levels of VEGF and TGFβ in the mouse models with or without tumor. **(F)** The positive rates of IFNα and GM-CSF. N, the tumor-free mice; T, the mice with tumor.

Supplementary Figure 4 Effects of HBx mutations on malignant phenotypes of cancer cells. **(A)** The overexpression of WT-HBx and HBx mutants in HepG2 and HeLa cells. **(B)** Ectopic expression of the HBx mutants had no significant effect on the invasion of HepG2 and HeLa cells, compared to WT-HBx. **(C)** Representative images of the transwell assays for cell migration in HepG2 cells. **(D)** Representative images of the transwell assays for cell invasion in HepG2 cells.

Supplementary Figure 5 Protein-protein interaction (PPI) networks and inflammatory gene set identified in cDNA microarray data. **(A)** PPI networks generated with the differential genes identified in M3-HBx expressing HeLa cells and Ct-HBx expressing HeLa cells. **(B)** CROONQUIST_IL6_DEPRIVATION_DN, the IL-6-related gene set, was significantly enriched in the cDNA microarray data of M3-HBx-injected mice. **(C)** CROONQUIST_IL6_DEPRIVATION_DN, the IL-6-related gene set, was significantly enriched in the cDNA microarray data of Ct-HBx-injected mice.

Supplementary Figure 6 The effects of HBx mutants on the promoter activities of *PAI1*, *CDC20*, and *P21.* **(A)** The results of luciferase assays in HepG2 cells. **(B)** The results of luciferase assays in HeLa cells.

## Abbreviations

PLC, Primary liver cancer; HCC, hepatocellular carcinoma; HBV, hepatitis B virus; HBsAg, HBV surface antigen; HBV-HCC, HBV-related HCC; AFP, alpha-fetoprotein; CP, core promoter; SKP2, S-phase kinase associated protein 2; HBx, hepatitis B virus X; Ct-HBx, carboxylic acid-terminal truncated HBx; WT, wild-type; SB, Sleeping Beauty; Fah, fumaryl acetoacetate hydrolase; STR, short tandem repeat; siRNAs, Small interfering RNAs; CDC20, cell division cycle 20; PAI1, plasminogen activator inhibitor-1; P21(CDKN1A), cyclin dependent kinase inhibitor 1A; qRT-PCR, real-time quantitative reverse transcription PCR; NTBC, 2-(2-nitro-4-trifluoromethylbenzoyl)-1,3-cyclohexanedione; H&E, hematoxylin-eosin; SRA, Sequence Read Archive; IL-4, interleukin-4; IFN-α, interferon-α; TNF-α, tumor necrosis factor-α; GM-CSF, granulocyte macrophage colony stimulating factor; TGFβ, transforming growth factor β; VEGF, vascular endothelial growth factor; RNA-seq, RNA-sequencing; GSEA, gene set enrichment analysis; GEO, Gene Expression Omnibus; M1, A1762T/G1764A; M2, T1674G+T1753C+A1762T/G1764A; M3, C1653T+T1674G+A1762T/G1764A; IHC, Immunohistochemistry; CK18, cytokeratin 18; Ctnna3, catenin alpha 3; Fhit, fragile histidine triad gene; TERT, telomerase reverse transcriptase; Th1, T helper 1; PPI, protein-protein interaction.
