# Supplementary figures and images for: The Effects and Underlying Mechanisms of Hepatitis B Virus X Gene Mutants on the Development of Hepatocellular Carcinoma
Source: Front Oncol. 2022 Feb 10;12:836517. doi: 10.3389/fonc.2022.836517 (PMC8867042; doi:10.3389/fonc.2022.836517)

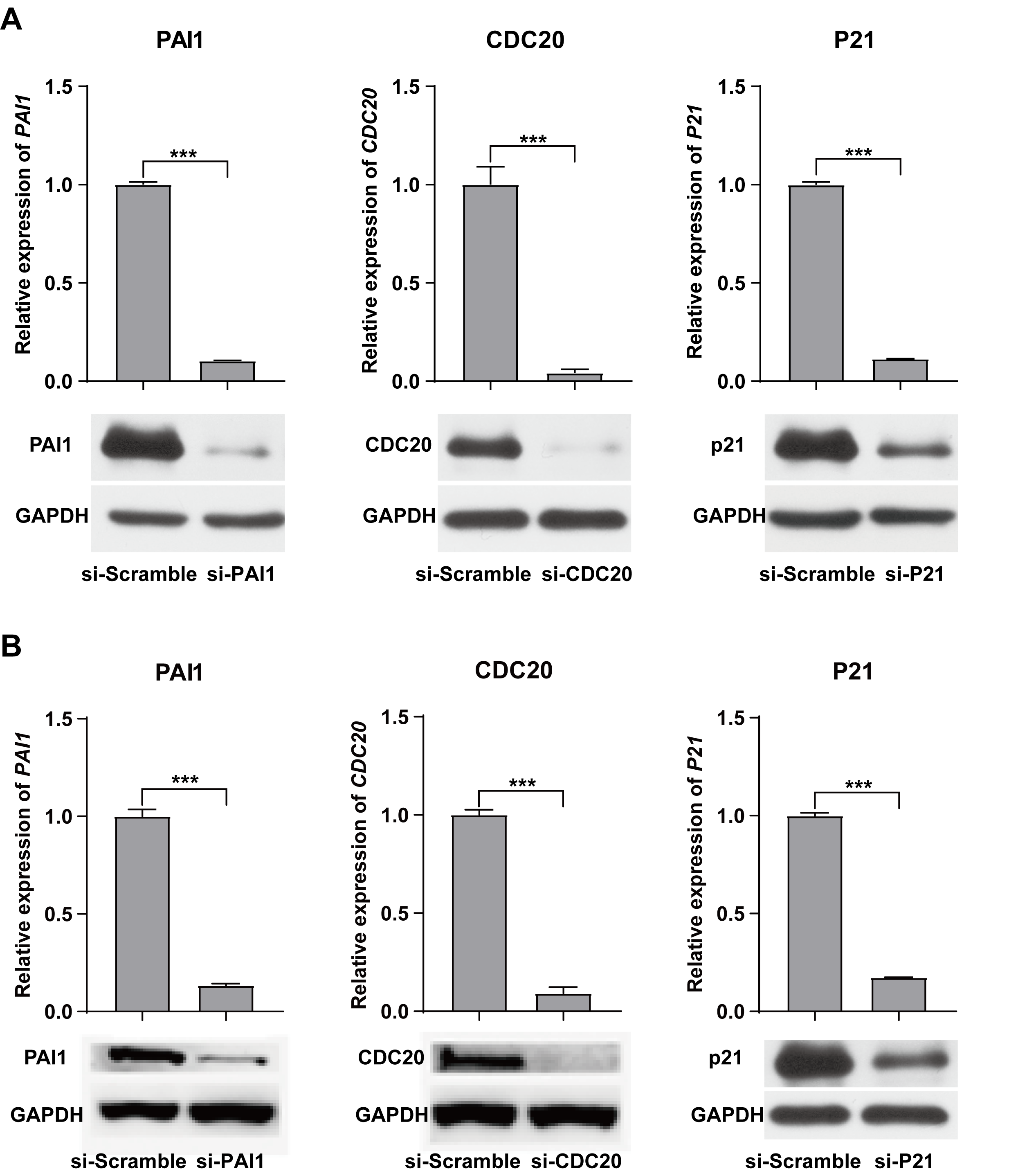

Supplement: Supplementary Figure 1 — The efficiency of gene knockdown. (A) The mRNA and protein levels of CDC20, PAI1, and p21 in HepG2 cells. (B) The mRNA and protein levels of CDC20, PAI1, and p21 in HeLa cells. ***P < 0.001. [file Image_1.tif]

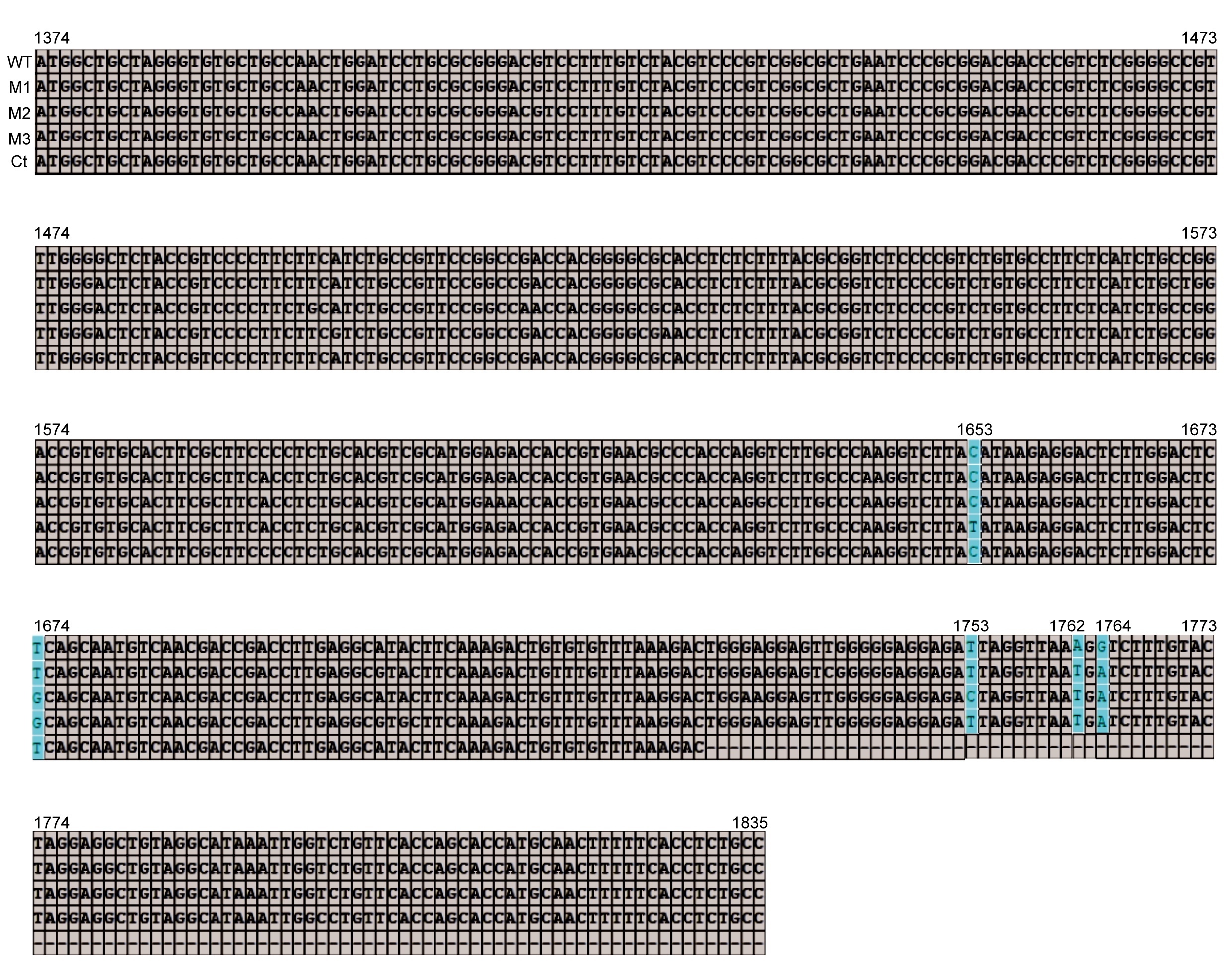

Supplement: Supplementary Figure 2 — The sequences of HBx fragments amplified from the peripheral serum samples of HBV-infected patients. WT, the HBx fragment without HCC-related mutations; M1, the HBx fragment carrying A1762T/G1764A; M2, the HBx fragment carrying combo mutation A1762T/G1764A+T1674G+T1753C; M3, the HBx fragment carrying combo mutation C1653T+T1674G+A1762T/G1764A; Ct, the fragment of carboxylic acid-terminal truncated HBx. [file Image_2.tif]

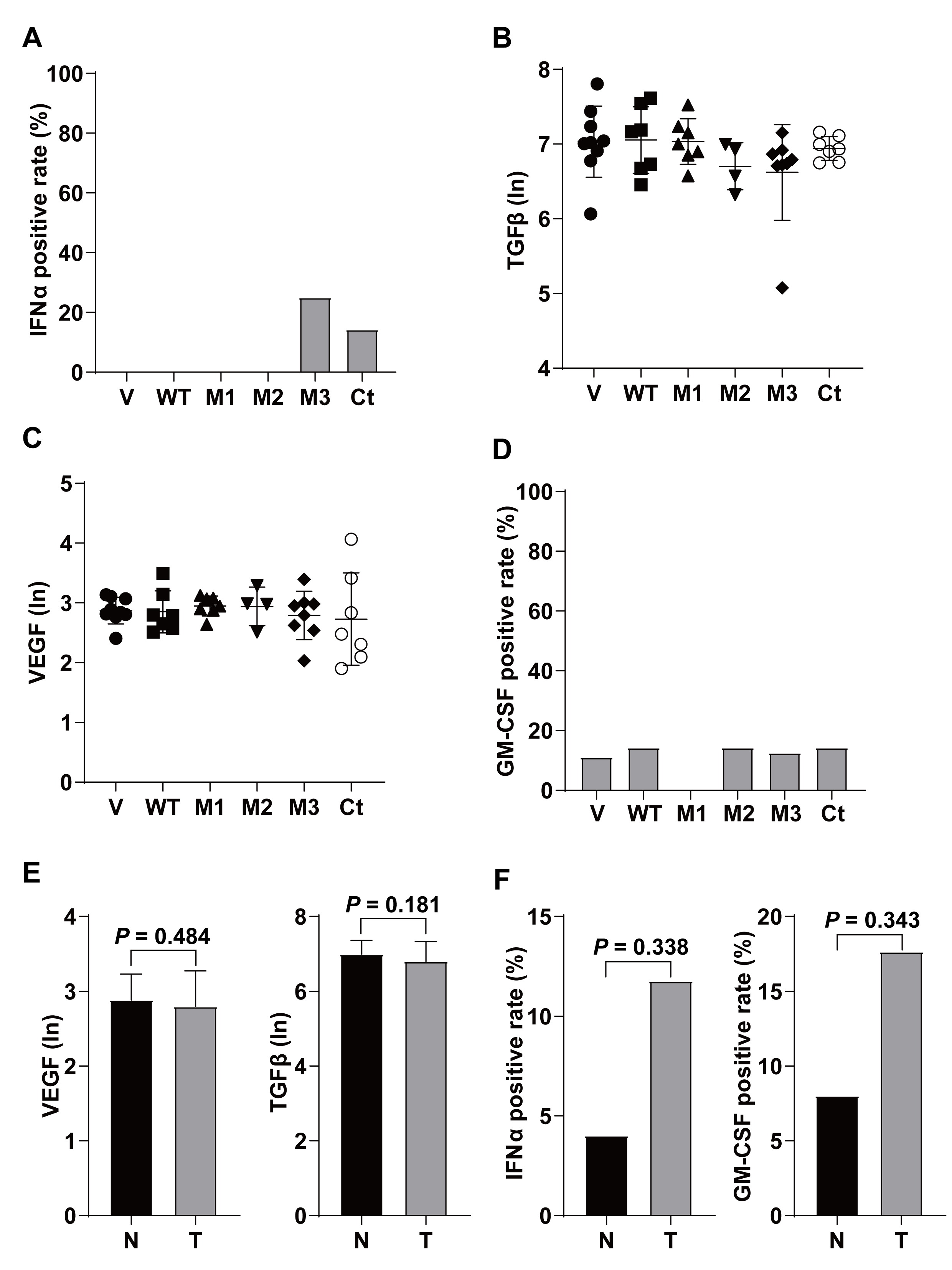

Supplement: Supplementary Figure 3 — The serum levels and the positive rates of cytokines in the SB mouse models. (A) The serum level of VEGF. (B) The serum level of TGFβ. (C) The positive rate of IFNα. (D) The positive rate of GM-CSF. (E) The serum levels of VEGF and TGFβ in the mouse models with or without tumor. (F) The positive rates of IFNα and GM-CSF. N, the tumor-free mice; T, the mice with tumor. [file Image_3.tif]

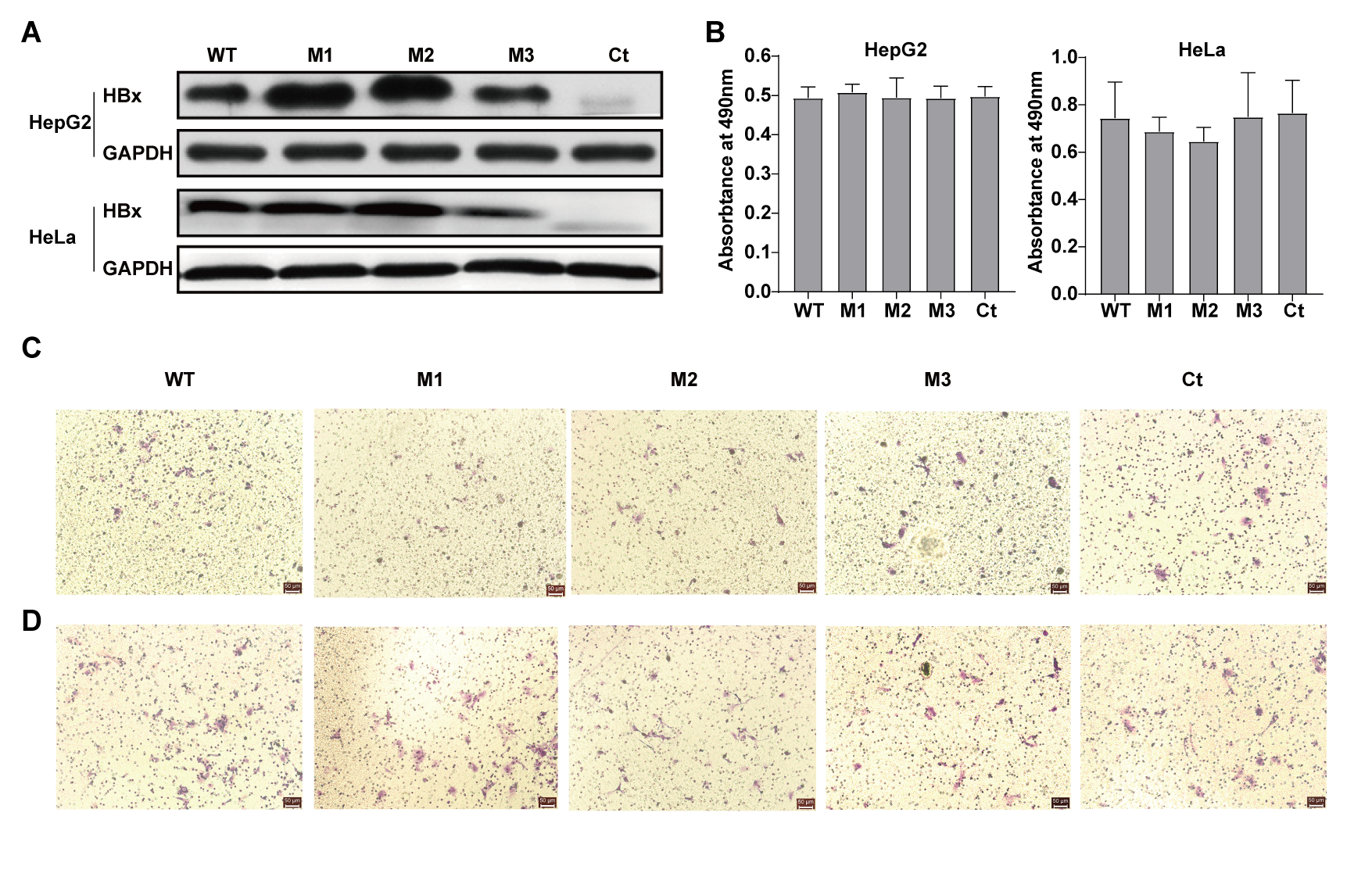

Supplement: Supplementary Figure 4 — Effects of HBx mutations on malignant phenotypes of cancer cells. (A) The overexpression of WT-HBx and HBx mutants in HepG2 and HeLa cells. (B) Ectopic expression of the HBx mutants had no significant effect on the invasion of HepG2 and HeLa cells, compared to WT-HBx. (C) Representative images of the transwell assays for cell migration in HepG2 cells. (D) Representative images of the transwell assays for cell invasion in HepG2 cells. [file Image_4.tif]

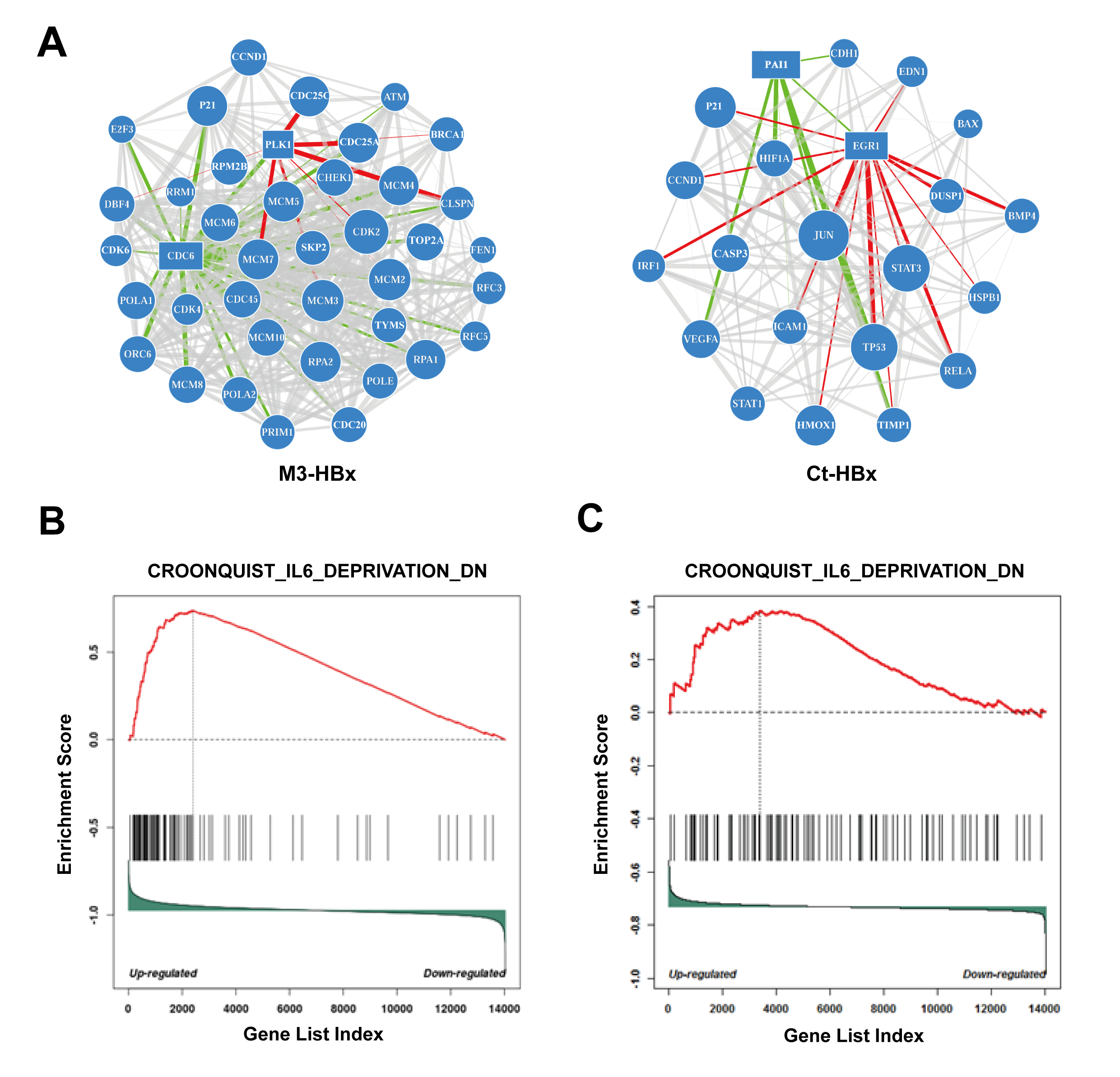

Supplement: Supplementary Figure 5 — Protein-protein interaction (PPI) networks and inflammatory gene set identified in cDNA microarray data. (A) PPI networks generated with the differential genes identified in M3-HBx expressing HeLa cells and Ct-HBx expressing HeLa cells. (B) CROONQUIST_IL6_DEPRIVATION_DN, the IL-6-related gene set, was significantly enriched in the cDNA microarray data of M3-HBx-injected mice. (C) CROONQUIST_IL6_DEPRIVATION_DN, the IL-6-related gene set, was significantly enriched in the cDNA microarray data of Ct-HBx-injected mice. [file Image_5.tif]

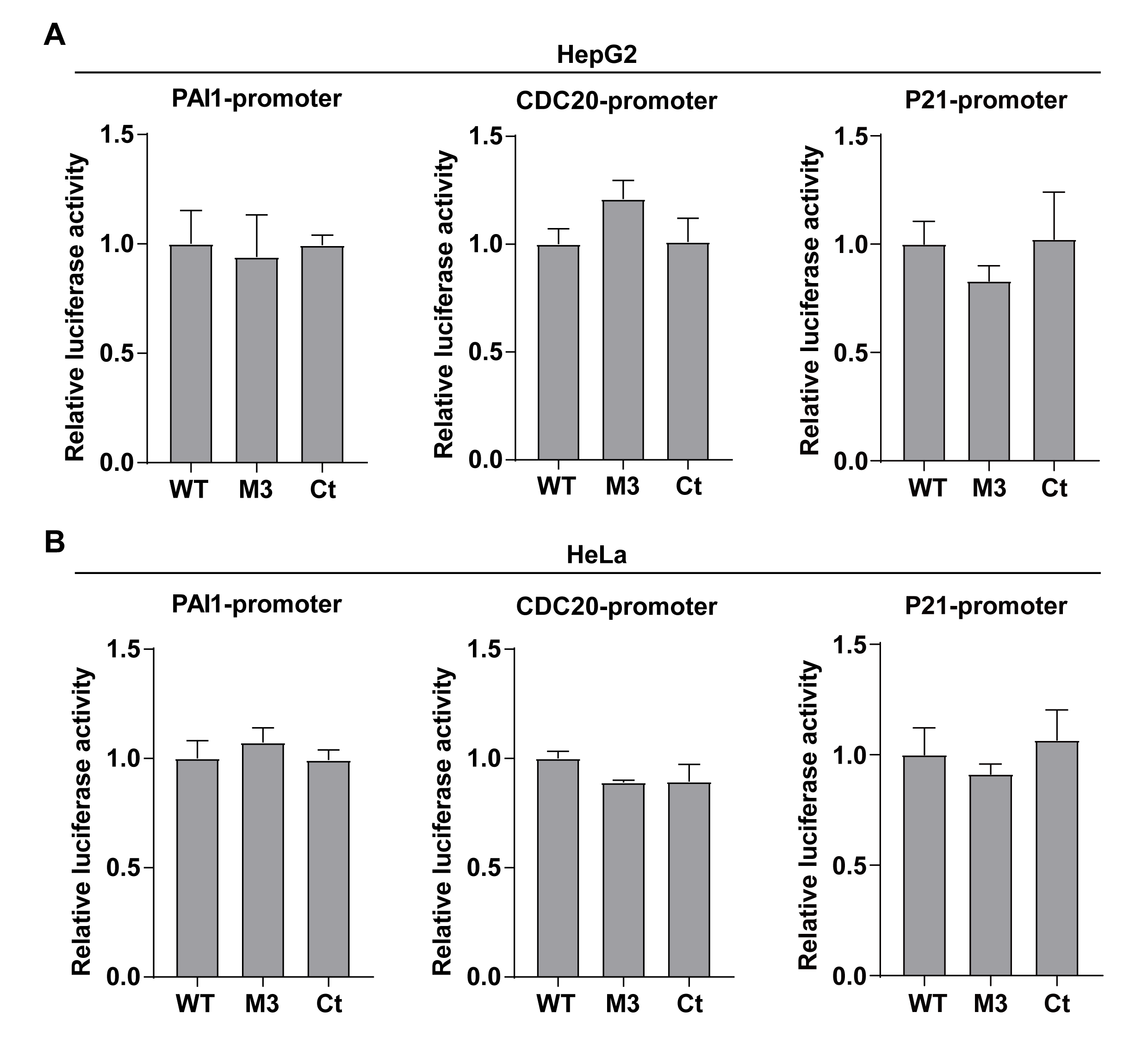

Supplement: Supplementary Figure 6 — The effects of HBx mutants on the promoter activities of PAI1, CDC20, and P21. (A) The results of luciferase assays in HepG2 cells. (B) The results of luciferase assays in HeLa cells. [file Image_6.tif]
