# Supplementary material for: The Effects and Underlying Mechanisms of Hepatitis B Virus X Gene Mutants on the Development of Hepatocellular Carcinoma
Source: Front Oncol. 2022 Feb 10;12:836517. doi: 10.3389/fonc.2022.836517 (PMC8867042; doi:10.3389/fonc.2022.836517)
Supplement: Supplementary file 7 [file DataSheet_1.docx]

**Supplementary material**

**The effects and underlying mechanisms of hepatitis B virus X gene mutants on the development of hepatocellular carcinoma**

**Supplementary methods**

*Extraction of viral DNA*

Five ml fasting blood from all participants was collected with a vacuum blood collection tube without anticoagulants. The serum was separated by centrifugation at 4°C. HBV DNA was extracted from 200 ml sera by using the serum viral DNA purification kit (PG Biotechnology, Shenzhen, China).

*HBV DNA sequencing and mutation analysis*

The HBV X gene (nt.1374-nt.1838) was amplified by PCR. The primers are listed in Table S1. PCR was performed in 25μl mixture containing 3μl diluted HBV DNA, 1×PCR buffer, 1.5U Taq polymerase (TaKaRa Biotechnology, Dalian, China), 0.2μM each primer (synthesized by Sangon Biotechnology, Shanghai, China), and 0.2mM dNTP (TaKaRa). The amplification was performed with an annealing temperature of 58°C for 60s for 35 cycles by using an Autorisierter thermocycler (Eppendorf AG, Hamburg, Germany). DNA was gel purified and sequenced by Sanger sequencing (Maipu, Shanghai, China). Sequence alignment and analysis were performed by using MEGA 4.0 software. The sequence of the wild-type HBx was identified by our previous study.^(1)^

*Construction of recombinant lentivirus expressing WT-HBx and HBx mutants*

The sequences of the wild-type and four mutant HBx fragments were synthesized by Obio Technology (Shanghai, China) and were coloned into the plteni-CMV-GFP-puro lentiviral vector by GeneArt Seamless Cloning and Assembly Kit (Thermo Fisher Scientific, MA). Lentiviruses were packaged using Lentiviral Packaging System (Invitrogen, Carlsbad, CA) according to the manufacture’s instruction. Briefly, the constructed lentiviral vectors were transfected into HEK293T cells together with the MD2G packaging plasmid and PAX2 envelope plasmid by using Lipofectamine 3000 kit (Invitrogen). After incubation for 48h, lentivirus-containing supernatants were harvested and lentiviral particles were concentrated using Lenti-X (Clontech, CA).

*Cell culture and the stable transfection of HBx variants*

HepG2 and HeLa cell lines were purchased from the Chinese Academy of Sciences (Shanghai, China). All cells were cultured in Dulbecco’s modified Eagle’s medium (DMEM, HyClone, Logan, UT) with 10% fetal bovine serum (Gibco, New York, NY) and 1% penicillin/streptomycin (Bio-light, Shanghai, China). Cells were infected with the overexpression lentivirus and the negative control lentivirus for 24h. The cell stains with the stable expression of HBx protein were screened by 20μg/mL blasticidin (Thermo Fisher, Waltham, MA).

*Real-time quantitative reverse transcription (qRT-PCR) and Western blot*

Total RNA was extracted, reversely transcribed into cDNA, and used as the template for qRT-PCR as previously described.^(2)^ Gene expression was relatively quantified using glyceraldehyde-3-phosphate dehydrogenase (*GAPDH*) as an internal control. The primers for qRT-PCR are listed in Table S1. Protein was extracted, quantified, and subjected to Western blot as previously described.^(2)^ The primary antibodies were listed in Table S3. GAPDH was used as the loading control. ImageJ software (version 1.51, https://imagej.nih.gov/ij/) was applied to quantify the signal strength of each band.

*Immunohistochemistry (IHC)*

Tissue samples were 10% formalin-fixed and paraffin-embedded for hematoxylin-eosin (H&E) and immunohistochemistry analyses as previously described.^(3)^ Antibodies used were detailed in Table S3. The H&E slides were reviewed by a pathologist (Jianfeng Wu). IHC scores were independently assessed by three investigators who were blind to the experimental data. The staining results were assessed by semi-quantitative methods including staining intensity (0, negative; 1, low; 2, moderate; 3, strong) and the score for the proportion of stained area (0, <10%; 1, 10%-30%; 2, 31%-60%; 3, >60%). The intensity and area of IHC staining were assessed in 5 visual fields. The final IHC score was calculated with the formula: (staining intensity scores in 5 visual fields + staining area scores in 5 visual fields )/5. Disagreements were resolved by consensus.

**References:**

1. Jianhua Yin, Jiaxin Xie, Shijian Liu, Hongwei Zhang, Lei Han, Wenying Lu, et al. Association between the various mutations in viral core promoter region to different stages of hepatitis B, ranging of asymptomatic carrier state to hepatocellular carcinoma. Am J Gastroenterol 2011;106:81-92.
2. Li X, Tan X, Yu Y, Chen H, Chang W, Hou J, et al. D9S168 microsatellite alteration predicts a poor prognosis in patients with clear cell renal cell carcinoma and correlates with the down-regulation of protein tyrosine phosphatase receptor delta. Cancer. 2011;117:4201-4211.
3. Chang W, Gao X, Han Y, Du Y, Liu Q, Wang L, et al. Gene expression profiling-derived immunohistochemistry signature with high prognostic value in colorectal carcinoma. Gut 2014;63:1457-1467.

**Legends of supplementary figures**

**Figure S1. The efficiency of gene knockdown**

(A) The mRNA and protein levels of CDC20, PAI1, and p21 in HepG2 cells. (B) The mRNA and protein levels of CDC20, PAI1, and p21 in HeLa cells. *** *P <* 0.001.

**Figure S2. The sequences of HBx fragments amplified from the peripheral serum samples of HBV-infected patients**

WT, the HBx fragment without HCC-related mutations; M1, the HBx fragment carrying A1762T/G1764A; M2, the HBx fragment carrying combo mutation A1762T/G1764A+T1674G+T1753C; M3, the HBx fragment carrying combo mutation C1653T+T1674G+A1762T/G1764A; Ct, the fragment of carboxylic acid-terminal truncated HBx.

**Figure S3. The serum levels and the positive rates of cytokines in the SB mouse models**

(A) The serum level of VEGF. (B) The serum level of TGFβ. (C) The positive rate of IFNα. (D) The positive rate of GM-CSF. (E) The serum levels of VEGF and TGFβ in the mouse models with or without tumor. (F) The positive rates of IFNα and GM-CSF. N, the tumor-free mice; T, the mice with tumor.

**Figure S4. Effects of HBx mutations on malignant phenotypes of cancer cells**

(A) The overexpression of WT-HBx and HBx mutants in HepG2 and HeLa cells. (B) Ectopic expression of the HBx mutants had no significant effect on the invasion of HepG2 and HeLa cells, compared to WT-HBx. (C) Representative images of the transwell assays for cell migration in HepG2 cells. (D) Representative images of the transwell assays for cell invasion in HepG2 cells.

**Figure S5. Protein-protein interaction (PPI) networks and inflammatory gene set identified in cDNA microarray data**

(A) PPI networks generated with the differential genes identified in M3-HBx expressing HeLa cells and Ct-HBx expressing HeLa cells. (B) CROONQUIST_IL6_DEPRIVATION_DN, the IL-6-related gene set, was significantly enriched in the cDNA microarray data of M3-HBx-injected mice. (C) CROONQUIST_IL6_DEPRIVATION_DN, the IL-6-related gene set, was significantly enriched in the cDNA microarray data of Ct-HBx-injected mice.

**Figure S6. The effects of HBx mutants on the promoter activities of *PAI1*, *CDC20*, and *P21***

**(**A) The results of luciferase assays in HepG2 cells. **(**B) The results of luciferase assays in HeLa cells.
