# Supplementary material for: The Effects and Underlying Mechanisms of Hepatitis B Virus X Gene Mutants on the Development of Hepatocellular Carcinoma
Source: Front Oncol. 2022 Feb 10;12:836517. doi: 10.3389/fonc.2022.836517 (PMC8867042; doi:10.3389/fonc.2022.836517)
Supplement: Supplementary file 8 [file Table_1.doc]

**Table S1. Primers for the amplification and verification.**

| Purpose | Sequence |
| --- | --- |
| for HBx PCR (nt.1060-1838) | Forward 5’-TTATATGCATGTATACAATC-3’ |
|  | Reverse 5’-TTAGGCAGAGGTGAAAAAGTTGC-3’ |
| for pKT2-FAH-mCa-SB-HBX construction (nt.1374-1838) | Forward 5’-CCGGAATTCTTATATGCATGTATACAATC-3’ |
|  | Reverse 5’-CCGGAATTCTTAGGCAGAGGTGAAAAAGTTGC-3’ |
| for pKT2-FAH-mCa-SB-HBX construction (nt.1374-1733) | Forward 5’-CCGGAATTCTTATATGCATGTATACAATC-3’ |
|  | Reverse 5’-CCGGAATTCTCAGTCTTTAAACACACAGTCTTTGA-3’ |
| for *PAI1* RT-qPCR | Forward 5’-CGCTGTCAAGAAGACCCACA-3’ |
|  | Reverse 5’-AGTTCTCAGAGGTGCCTTGC-3’ |
| for *CDC20* RT-qPCR | Forward 5’-GCACAGTTCGCGTTCGAGA-3’ |
|  | Reverse 5’-CTGGATTTGCCAGG AGTTCGG-3’ |
| for *P21* RT-qPCR | Forward 5’-TGTCCGTCAGAACCCATGC-3’ |
|  | Reverse 5’-AAAGTCGAAGTTCCATCGCTC-3’ |
| for *SKP2* RT-qPCR | Forward 5’-ATGCCCCAATCTTGTCCATCT-3’ |
|  | Reverse 5’-CACCGACTGAGTGATAGGTGT-3’ |
