# Supplementary material for: The Effects and Underlying Mechanisms of Hepatitis B Virus X Gene Mutants on the Development of Hepatocellular Carcinoma
Source: Front Oncol. 2022 Feb 10;12:836517. doi: 10.3389/fonc.2022.836517 (PMC8867042; doi:10.3389/fonc.2022.836517)
Supplement: Supplementary file 9 [file Table_2.doc]

**Table S2. The sequences of siRNA.**

| Target Gene | Sequence 5’→3’ |
| --- | --- |
| CDC20 | CGGAAGACCTGCCGTTACA |
| PAI1 | CTATGGGATTCAAGATTGA |
| CDKN1A (P21) | GGACCTGTCACTGTCTTGT |
