# Supplementary material for: The Effects and Underlying Mechanisms of Hepatitis B Virus X Gene Mutants on the Development of Hepatocellular Carcinoma
Source: Front Oncol. 2022 Feb 10;12:836517. doi: 10.3389/fonc.2022.836517 (PMC8867042; doi:10.3389/fonc.2022.836517)
Supplement: Supplementary file 10 [file Table_3.doc]

**Table S3. Antibodies for Western blot and Immunohistochemistry**

| Proteins | Antibodies | Source | Catalog number | Dilution ratio | References |
| --- | --- | --- | --- | --- | --- |
| CDC20  (for western blot) | CDC20 (D6C2Q) Rabbit mAb | Cell Signaling | 14866 | 1:1000 | Zhaowei Chu, et. al. CDC20 contributes to the development of human cutaneous squamous cell carcinoma through the Wnt/β‑catenin signaling pathway.  Int J Oncol. 2019 May;54(5):1534-1544. |
| PAI1  (for western blot) | Rabbit Anti-PAI1 antibody | Abcam | ab66705 | 1:1000 | Liu S et al. Transplantation of adipose tissue lacking PAI-1 improves glucose tolerance and attenuates cardiac metabolic abnormalities in high-fat diet-induced obesity. Adipocyte 9:170-178 (2020). |
| p21  (for western blot) | p21 Waf1/Cip1 (12D1) Rabbit mAb | Cell Signaling | 2947 | 1:1000 | Yi-Deun Jung, et. al. Epigenetic regulation of miR-29a/miR-30c/DNMT3A axis controls SOD2 and mitochondrial oxidative stress in human mesenchymal stem cells.  Redox Biol 2020 Sep 9;37:101716. |
| SKP2  (for western blot) | Skp2 (D3G5) XP® Rabbit mAb | Cell Signaling | 2652 | 1:1000 | Jichuan Wang, et. al. Skp2 depletion reduces tumor-initiating properties and promotes apoptosis in synovial sarcoma. Transl Oncol 2020 Oct;13(10):100809. |
| GAPDH  (for western blot) | Anti-GAPDH antibody | SantaCruz | sc-47724 | 1:2000 | Leung, EL. et al. Identification of a new inhibitor of KRAS-PDEδ interaction targeting KRAS mutant nonsmall cell lung cancer. 2019. Int. J. Cancer. |
| Flag  (for western blot) | ANTI-FLAG® antibody | Sigma | F7425 | 1:1000 | Yixing Li et. al. The dynamics of FTO binding and demethylation from the m6A motifs. RNA biology, 16(9), 1179-1189 (2019-6-1) |
| HBxAg  (for IHC) | Hepatitis B x Antigen (HBxAg) Mouse anti-Virus Monoclonal (3F6-G10) Antibody | LifeSpan BioSciences | LS‑C57338 | 1:100 | There are no publications for anti-HBxAg antibody (LS‑C57338). |
| CK-18  (for IHC) | Anti-Cytokeratin 18 antibody [EPR17347] | Abcam | ab181597 | 1:800 | Zhang S et al. Platelet-rich plasma improves therapeutic effects of menstrual blood-derived stromal cells in rat model of intrauterine adhesion. Stem Cell Res Ther 10:61 (2019). |
| AFP  (for IHC) | Anti-AFP antibody produced in rabbit | Sigma | SAB3500533 | 1:200 | Wenwen Deng et. al. Pleurotus eryngii Polysaccharide Promotes Pluripotent Reprogramming via Facilitating Epigenetic Modification. Journal of agricultural and food chemistry, 64(6), 1264-1273 (2016-1-27) |
| CDC20  (for IHC) | Anti-Cdc20 antibody | Abcam | ab86104 | 1:250 | Huo X et al. Identification of prognosis markers for endometrial cancer by integrated analysis of DNA methylation and RNA-Seq data. Sci Rep 9:9924 (2019). |
| PAI1  (for IHC) | Rabbit Anti-PAI1 antibody | Abcam | ab66705 | 1:200 | Eriksson BO et al. Upregulation of Plasminogen Activator Inhibitor-1 in Irradiated Recipient Arteries and Veins from Free Tissue Transfer Reconstruction in Cancer Patients. Mediators Inflamm 2018:4058986 (2018). |
| p21  (for IHC) | Anti-p21 antibody [EPR18021] | Abcam | ab188224 | 1:200 | Desamero MJ et al. Tumor-suppressing potential of stingless bee propolis in in vitro and in vivo models of differentiated-type gastric adenocarcinoma. Sci Rep 9:19635 (2019). |
