# Supplementary material for: The Effects and Underlying Mechanisms of Hepatitis B Virus X Gene Mutants on the Development of Hepatocellular Carcinoma
Source: Front Oncol. 2022 Feb 10;12:836517. doi: 10.3389/fonc.2022.836517 (PMC8867042; doi:10.3389/fonc.2022.836517)
Supplement: Supplementary file 11 [file Table_4.doc]

**Table S4. Distribution of HBV integration sites**

| Group | Sample-ID | Total number^*^ | Intergenic region | Intron | Exon |
| --- | --- | --- | --- | --- | --- |
| WT-LIVER | WT-1 | 72 | 40 | 32 | 1 |
| WT-LIVER | WT-2 | 67 | 33 | 33 | 2 |
| WT-LIVER | WT-3 | 641 | 362 | 263 | 17 |
| WT-CANCER | WT-4T | 38 | 25 | 13 | 0 |
| M3-LIVER | M3-1 | 101 | 63 | 34 | 4 |
| M3-CANCER | M3-2T | 98 | 58 | 36 | 5 |
| M3-CANCER | M3-3T | 101 | 63 | 34 | 4 |
| M3-CANCER | M3-4T | 25 | 11 | 13 | 1 |
| Ct-LIVER | Ct-1 | 145 | 85 | 54 | 7 |
| Ct-LIVER | Ct-2 | 78 | 46 | 22 | 10 |
| Ct-LIVER | Ct-3 | 137 | 81 | 53 | 4 |
| Ct-CANCER | Ct-4T | 27 | 16 | 11 | 1 |
| Ct-CANCER | Ct-5T | 215 | 120 | 85 | 11 |
| Ct-CANCER | Ct-6T | 5 | 2 | 3 | 0 |

^*^ The total number may smaller than the sum of all groups. Because some insertion site belong to the different transcript variants.
