# Supplementary material for: The Effects and Underlying Mechanisms of Hepatitis B Virus X Gene Mutants on the Development of Hepatocellular Carcinoma
Source: Front Oncol. 2022 Feb 10;12:836517. doi: 10.3389/fonc.2022.836517 (PMC8867042; doi:10.3389/fonc.2022.836517)
Supplement: Supplementary file 12 [file Table_5.docx]

**Table S5. Frequently integrated genes of the sleeping beauty mice models^*^**

| **Gene** | **Frequency of integration** | **Functional region** | **Integration depth**^†^  **(median)** | **Sample-ID** |
| --- | --- | --- | --- | --- |
| *Fah* | 6/14 | Intron, exon | 0.12% | WT-1,WT-3,M3-2T,M3-3T,CT-1,CT-2 |
| *Ctnna3* | 4/14 | Intron | 0.21% | WT-3,M3-2T,M3-3T,CT-5T |
| *Fhit* | 4/14 | Intron | 0.16% | WT-3,M3-2T,M3-4T,CT-5T |
| *Nrxn3* | 3/14 | Intron | 0.11% | WT-1,WT-2,M3-1 |
| *Csmd1* | 3/14 | Intron | 0.14% | WT-3,CT-1,CT-3 |
| *Dmd* | 3/14 | Intron | 0.23% | WT-3,CT-1,CT-5T |
| *Epha6* | 3/14 | Intron | 0.25% | WT-3,CT-1,CT-5T |
| *Naaladl2* | 3/14 | Intron | 0.09% | WT-2,M3-2T,M3-3T |
| *Stk24* | 3/14 | Intron | 0.09% | CT-2,CT-4T,CT-5T |

**^*^**Frequently integrated gene was defined as the gene with the integration events detected in more than three samples.

^†^The ratio of the reads with integration to the normal reads.
