# Supplementary material for: The Effects and Underlying Mechanisms of Hepatitis B Virus X Gene Mutants on the Development of Hepatocellular Carcinoma
Source: Front Oncol. 2022 Feb 10;12:836517. doi: 10.3389/fonc.2022.836517 (PMC8867042; doi:10.3389/fonc.2022.836517)
Supplement: Supplementary file 13 [file Table_6.docx]

**Table S6. Key molecules in the protein-protein interaction (PPI) networks.**

| Gene | Differential expression analysis in *sleeping beauty* mice^1^ | | |
| --- | --- | --- | --- |
|  | Group comparison | Fold change | P |
| PAI1 | M3-HBx vs. WT-HBx  Ct-HBx vs. WT-HBx | 16.93 | <0.0001 |
|  |  | 3.37 | 0.00188 |
|  |  |  |  |
| CDC20 | M3-HBx vs. WT-HBx | 2.64 | 0.00535 |
| P21 | M3-HBx vs. WT-HBx  Ct-HBx vs. WT-HBx | 3.27  1.78 | 0.00682  0.00912 |
|  |  |  |  |
| SKP2 | M3-HBx vs. WT-HBx | 1.66 | 0.04241 |
|  | Ct-HBx vs. WT-HBx | 1.68 | 0.04325 |

^1.^The expression of key molecules in the PPI network was further investigated with the microarray data of sleeping beauty models.
