# Supplementary material for: The Effects and Underlying Mechanisms of Hepatitis B Virus X Gene Mutants on the Development of Hepatocellular Carcinoma
Source: Front Oncol. 2022 Feb 10;12:836517. doi: 10.3389/fonc.2022.836517 (PMC8867042; doi:10.3389/fonc.2022.836517)
Supplement: Supplementary file 14 [file Table_7.docx]

**Table S7. The cancer-related gene sets that includes hub genes and was enriched in both HepG2 cells and *SB* mice.**

| Gene set | Included hub gene^*^ | Group | FDR | | |
| --- | --- | --- | --- | --- | --- |
|  |  |  | HepG2 | mice | HeLa |
| ELVIDGE_HYPOXIA_BY_DMOG_UP | PAI1 | M3 | <0.0001 | <0.0001 | 0.2052 |
| ELVIDGE_HYPOXIA_UP | PAI1 | M3 | <0.0001 | <0.0001 | 0.0507 |
| BORLAK_LIVER_CANCER_EGF_UP | PAI1 | M3 | 0.0387 | <0.0001 | 0.0575 |
| CHARAFE_BREAST_CANCER_LUMINAL_VS_MESENCHYMAL_DN | PAI1 | M3 | 0.0252 | <0.0001 | - |
| CROMER_TUMORIGENESIS_UP | PAI1 | M3 | 0.0127 | 0.0017 | 0.0326 |
| GRUETZMANN_PANCREATIC_CANCER_UP | PAI1 | M3 | 0.0038 | 0.0002 | 0.0005 |
| HINATA_NFKB_TARGETS_FIBROBLAST_UP | PAI1 | M3 | 0.0119 | 0.0027 | - |
| HINATA_NFKB_TARGETS_KERATINOCYTE_UP | PAI1 | M3 | 0.0113 | 0.0005 | 0.0326 |
| NAKAYAMA_SOFT_TISSUE_TUMORS_PCA1_UP | PAI1 | M3 | <0.0001 | 0.0010 | - |
| SWEET_KRAS_TARGETS_UP | PAI1 | M3 | 0.0024 | 0.0009 | 0.0215 |
| SWEET_LUNG_CANCER_KRAS_UP | PAI1 | M3 | 0.0043 | 0.0033 | <0.0001 |
| VERHAAK_GLIOBLASTOMA_MESENCHYMAL | PAI1 | M3 | <0.0001 | <0.0001 | 0.0094 |
| WANG_ESOPHAGUS_CANCER_VS_NORMAL_UP | PAI1 | M3 | 0.0445 | 0.0003 | 0.0268 |
| WU_CELL_MIGRATION | PAI1 | M3 | <0.0001 | <0.0001 | <0.0001 |
| YAMASHITA_METHYLATED_IN_PROSTATE_CANCER | PAI1 | M3 | <0.0001 | 0.0011 | - |
| NAKAMURA_CANCER_MICROENVIRONMENT_DN | CDC20 | M3 | 0.0467 | 0.0005 | - |
| NAKAYAMA_SOFT_TISSUE_TUMORS_PCA2_UP | CDC20 | M3 | 0.0029 | <0.0001 | 0.1062 |
| PUJANA_BREAST_CANCER_WITH_BRCA1_MUTATED_UP | CDC20, SKP2 | M3 | 0.0042 | <0.0001 | 0.0006 |
| ROSTY_CERVICAL_CANCER_PROLIFERATION_CLUSTER | CDC20 | M3 | <0.0001 | <0.0001 | <0.0001 |
| SCIAN_CELL_CYCLE_TARGETS_OF_TP53_AND_TP73_DN | CDC20 | M3 | 0.0065 | 0.0001 | 0.2449 |
| SOTIRIOU_BREAST_CANCER_GRADE_1_VS_3_UP | CDC20 | M3 | <0.0001 | <0.0001 | <0.0001 |
| TANG_SENESCENCE_TP53_TARGETS_DN | CDC20 | M3 | <0.0001 | <0.0001 | 0.0044 |
| CHIARADONNA_NEOPLASTIC_TRANSFORMATION_KRAS_CDC25_UP | P21 | M3 | 0.0415 | 0.0001 | 0.0864 |
| HENDRICKS_SMARCA4_TARGETS_UP | P21 | M3 | 0.0039 | 0.0053 | 0.0350 |
| VERNELL_RETINOBLASTOMA_PATHWAY_UP | SKP2 | M3 | 0.0127 | <0.0001 | <0.0001 |
| ZHANG_BREAST_CANCER_PROGENITORS_UP | SKP2 | M3 | 0.0405 | 0.0029 | <0.0001 |
| KERLEY_RESPONSE_TO_CISPLATIN_UP | PAI1, P21 | Ct | 0.0115 | 0.0028 | <0.0001 |

* Identified in PPI network analysis.
